# Development of Polyampholyte Cellulose-Based Hydrogels for Diapers with Improved Biocompatibility

**DOI:** 10.3390/gels11040282

**Published:** 2025-04-10

**Authors:** Beatriz Simões, Rafael C. Rebelo, Sara Ledesma, Patrícia Pereira, Rui Moreira, Brígida C. Ferreira, Jorge F. J. Coelho, Arménio C. Serra

**Affiliations:** 1 CEMMPRE, ARISE, Department of Chemical Engineering, University of Coimbra, Rua Sílvio Lima-Polo II, 3030-790 Coimbra, Portugal; 2 Instituto de Biofísica e Engenharia Biomédica, Faculdade de Ciências da Universidade de Lisboa, Campo Grande, 1749-016 Lisbon, Portugal; 3 Departamento de Ingeniería Química y Tecnologías del Medio Ambiente (IQTMA), Universidad de Zaragoza, C/María de Luna, 3., 50018 Zaragoza, Spain; 4 CERES, Department of Chemical Engineering, University of Coimbra, Rua Sílvio Lima-Polo II, 3030-790 Coimbra, Portugal; 5 IPN, Instituto Pedro Nunes, Associação para a Inovação e Desenvolvimento em Ciência e Tecnologia, Rua Pedro Nunes, 3030-199 Coimbra, Portugal

**Keywords:** allyl cellulose, cellulose copolymers, cellulose hydrogels, diapers, biocompatibility

## Abstract

Non-biodegradable superabsorbent polymers (SAPs) in personal care products (PCPs) pose significant environmental and health concerns despite their high absorption capacity. The aim of this study was to develop cellulose-based hydrogels as a sustainable alternative to those conventional SAPs, taking advantage of cellulose properties such as biocompatibility, biodegradability, and hydrophilicity. A synthesized allyl cellulose (AC) derivative was copolymerized with unusual monomers used in the production of SAPs, and the influence of monomer ratios, crosslinking density, and the ratio of cellulose to monomers on the absorption capacity was investigated and optimized. The most promising hydrogels were fully characterized for the proposed application and compared with a commercial SAP extracted from a baby diaper. The cellulose-based hydrogels showed promising absorption capacities in synthetic urine (~15 g/g), and a high centrifuge retention capacity (12.5 g/g), which was only slightly lower than the commercial SAP. These new hydrogels exhibited excellent biocompatibility and outperformed the established commercial diaper SAP. This study represents a more sustainable alternative to conventional SAPs, potentially reducing health risks while increasing the bio-based content of PCPs. Further optimization of these hydrogels could transform the hygiene product industry, by providing a balance between performance and environmental sustainability.

## 1. Introduction

The environmental impact of disposable products, in particular absorbent hygiene products (AHPs), such as baby diapers, feminine hygiene, and adult incontinence products, is increasingly recognized. These products are essential for the absorption of human fluids at different stages of life, and their use continues to increase, being indispensable in developed societies [1]. The high accumulation of AHPs in the environment and the potential toxicity of their constituents are currently a concern in environmental science and public health [2,3]. Even at minimal concentrations, the components of AHPs are capable of causing various physiological effects in the environment [4]. In addition to environmental problems, user health issues are also a concern. Diaper rash is a common condition associated with diapers, especially in children. Additionally, some commercially diapers contain dioxin-like compounds that are highly toxic and linked to cancer, developmental delays, weakened immunity, hormonal imbalances, and skin disorders [5]. Sodium polyacrylate, phthalates and heavy metals are chemicals found in diapers that can lead to adverse health effects. These products are commonly disposed of in landfills or incinerators. It is estimated that disposable diapers take approximately 500 years to decompose if landfilled [6,7]. The inadequate waste management of AHPs leads to the emission of non-biogenic CO_2_ and the persistent pollutants [8,9]. The environmental impact of these products can be mitigated by applying eco-design principles and innovative strategies.

Cellulose has been used as an absorbent component since the launch of AHPs. Due to its many hydrophilic hydroxyl groups, it can be easily wetted by water and aqueous solutions [10]. However, prior to the development of SAPs, a significant amount of absorbent cellulose pulp was required to absorb the same equivalent amount of urine. As a result, diapers were thicker, heavier, and had a significantly lower fluid absorption capacity. Those absorbent materials, such as cloth, cotton, paper wadding, and cellulose fibers, showed low efficiency and were prone to leakage [11,12]. Superabsorbent polymers hydrogels possess a three-dimensional (3D) hydrophilic structure that enables them to absorb and retain considerable amounts of aqueous solutions in their networks, being able to absorb more than 1000 times their dry weight [13,14] without dissolving [15]. Consequently, these polymers have transformed the personal care products industry by absorbing and retaining large amounts of liquid, even under pressure, ensuring the effectiveness of diapers and sanitary pads, while keeping the skin dry and healthy [16,17]. SAPs are a core component widely used in personal hygiene products, with disposable diapers being their main consumer. It is estimated that a baby uses about 4000 diapers, most of which are disposed in landfills, contributing to environmental pollution [12,17]. Furthermore, SAPs currently on the market are made from synthetic polymers, such as acrylic acid (AA) or acrylamide (AAm) and/or their derivatives [18]. Despite their exceptional ability to absorb and retain water and fluids, these polymers are neither bio-based nor biodegradable, which exacerbates the huge environmental impact [1,19].

The challenges associated with fossil-based hydrogels have led to an increased demand for bio-based superabsorbents that are more environmentally friendly and sustainable. One option is to replace conventional petroleum-based SAPs with bio-based materials, taking advantage of their biodegradability, biocompatibility, hydrophilicity, and non-toxicity [20]. This represents an environmentally conscious solution for personal care products and helps to reduce the dependence on fossil fuels and avoids greenhouse gas emissions. Among these bio-based materials, cellulose emerges as a particularly promising candidate as a main resource for the production of hydrogels used in personal care products [21,22]. Its excellent hydrophilic, biocompatible, and biodegradable properties provide a competitive advantage over the synthetic polymers currently used in those products [19]. Also, the properties desired for the applications can be achieved by functionalizing cellulose with active compounds [23]. As consumers increasingly prefer sustainable products, cellulose-based hydrogels are expected to experience significant growth in the hygiene products market in the future [24,25]. Cellulose-based hydrogels are being implemented in drug delivery systems, biomedical and personal health care applications, as well as in agriculture, horticulture, and the water treatment industry, as cellulose is abundant, safe, and mechanically stable [26].

Polyampholyte hydrogels represent a special class of hydrogels, characterized by the incorporation of both positively and negatively charged monomers [27]. These hydrogels offer several advantages over conventional hydrogels, including highly tunable properties such as mechanical, high pH and salt sensitivity, and non-fouling properties. They exhibit excellent biocompatibility and require minimal chemical crosslinking, as they rely instead on ionic interactions [28]. In combination with cellulose, they also exhibit high toughness and crack resistance. Since they react to environmental stimuli such as pH and salts presence, they can be used in several applications such as biomedical, soft electronics, or wastewater treatment. These properties make polyampholyte hydrogels superior materials for advanced biomedical and environmental technologies [29].

Although biodegradable absorbent products are a more sustainable alternative to commercially available options [30], they have not been industrialized yet. Currently, most of the cellulose used in health care consists of nonwoven cover, plastic film, and fluff absorbents of wood pulp cellulose. Despite advances, the commercialization of cellulose-based superabsorbents remains a challenge, primarily due to their swelling behavior and higher cost compared to conventional SAPs [31].

The aim of this study was to develop alternative cellulose-based hydrogels by leveraging the biological properties of cellulose. For such, an allyl cellulose derivative (AC) was synthesized and copolymerized with non-conventional monomers in SAPs to produce cellulose-based hydrogels. This was performed by the free radical polymerization (FRP) method, one of the simplest and most industrially used techniques to produce SAPs. The water and urine absorption capacities, salt and pH resistance, as well as the non-cytotoxicity properties of the prepared hydrogels were investigated. In addition, a comparative analysis between the most promising hydrogels and an SAP isolated from a commercially available baby diaper was performed to investigate potential practical applications.

## 2. Results and Discussion

### 2.1. Synthesis and Characterization of AC Derivative

**Figure 1:**
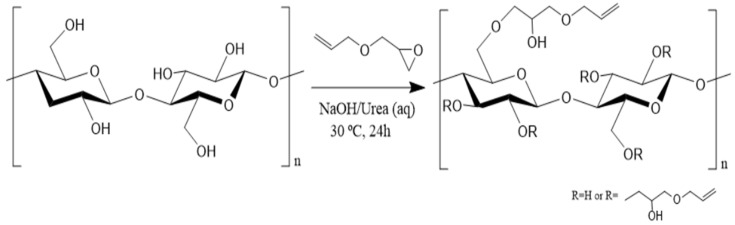
Chemical modification of cellulose with AGE in NaOH/urea/water solvent system.

The NaOH/urea/water solvent system limits the cellulose dissolution to a maximum molecular weight of the cellulose chains to less than 10^5^ g/mol, which corresponds to a maximum degree of polymerization (DP) of cellulose of approximately 600 [32]. Therefore, the DP of cellulose pulp was reduced to about 280 by acid hydrolysis, similar to those achieved by other hydrolysis methods [33,34]. This reduction in DP enabled the dissolution of cellulose and subsequent homogenous modification reaction. Cellulose can react directly with allyl glycidyl ether (AGE) in a NaOH/urea/water solvent system as the basicity of the solvent is enough to promote etherification [35]. Cellulose was modified with a ratio of 5:1 of AGE to anhydroglucose unity (AGU), as shown schematically in Figure 1, and the success of modification was evaluated by NMR and FTIR spectroscopies.

#### 2.1.1. NMR Spectroscopy of AC Derivative

The AC derivative was characterized by ^1^H- (Figure S1), ^13^C NMR (Figure S2), and ^1^H-^13^C HSQC (Figure S3) to confirm the presence of vinylic protons and determine the degree of substitution (DS). In ^1^H NMR spectra, the signals between 5.2 and 6.0 ppm (H3 and H4) confirmed the presence of the terminal vinyl groups in the cellulose derivatives. The signal observed between 4.4 and 4.6 ppm corresponded to the α-anomeric proton (H1) of the AGU. Additionally, peaks identified between 3.1 and 4.1 ppm were attributed to the remaining protons (AGU backbone, methylene protons of allyl substituents, and proton of the secondary alcohol formed by epoxy ring opening). The DS was calculated by dividing the integration area of the vinylic protons (H3 and H4) signals by the area of the anomeric proton signal (H1) [36,37,38]. The synthesized AC presented a DS of 1.13. Further confirmation of the functionalization of the cellulose was observed in ^13^C NMR spectra, where the characteristics peaks of the allylic carbon atoms were identified at 118.3 (C4) and 133.8 ppm (C3). Also, the signal at 72.0 (highest intensity peak in the agglomerate) is typical and attributed to the carbon atom C2 of the introduced functionalization. The signal at 102.3 ppm was assigned to the carbon of the α-anomeric proton (C1), and resultant signals from 55 to 85 ppm were assigned to the cellulose backbone and remaining carbon atoms of the introduced modification [35]. Further correlations between protons and carbon atoms were evaluated by 2D-NMR and ^1^H-^13^C HSQC. In the HSQC spectra, the correlation between H1 and C1 confirms the correct assignment of α-anomeric proton and carbon, the cross-over of the peaks between 5.0 and 6.0 ppm of H-NMR and the peaks at 115–135 ppm of the C-NMR confirms the presence of the carbons and attached protons of the double bonds. Also, a strong correlation between the protons (H2-4.0 ppm) and carbons (C2-72 ppm) of the methylene group which precedes the allyl group was found [35]. These correlations once again confirm the presence of allyl groups in cellulose derivatives.

#### 2.1.2. FTIR Spectroscopy of AC Derivative

Further confirmation of the synthesis of the AC derivative was obtained through Fourier-transform infrared spectroscopy (FTIR). The FTIR spectra of cellulose powder and AC derivative (see Section 2.3, FTIR spectra of dried samples of cellulose powder and AC derivative, respectively) exhibits the characteristic peaks of cellulosic materials such as the broad peak in the range of 3600–3100 cm^−1^, which corresponds to the stretching vibration of the hydrogen of the O-H bonds [39], besides the main characteristic peaks of the cellulose backbone: the peak at 900 cm^−1^ (β-glycosidic linkages), a broad peak at 1080 cm^−1^ (ring vibration and v C-O-C), at 1450 cm^−1^ (δ C-H), and at 2900 cm^−1^ (v C-H). The peak at 2900 cm^−1^ was separated into two distinct peaks in the cellulose derivatives spectrum, indicating structural modifications with respect to the stretching of C-H bonds [40,41]. The presence of allyl bonds in the AC sample was confirmed by the signal at 1650 cm^−1^, corresponding to the characteristic vibration of the C=C bond [42].

### 2.2. Preparation and Optimization of Cellulose-Based Hydrogels

Cellulose-based hydrogels were prepared by UV-FRP. The AC derivative and the designated monomer(s) and crosslinker were dissolved in deionized water, the UV initiator added, and irradiated for 2.5 h. This process aimed to implement a simple, affordable, and industrial-like process to prepare cellulose-based hydrogels. A chemically crosslinked hydrogel was obtained, and disk samples (Ø = 20 mm) were obtained and used in characterization and optimization studies.

**Figure 2:**
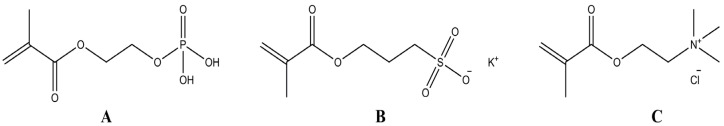
Chemical structure of the monomers used in this work: (**A**) phosphoric acid 2-hydroxyethyl methacrylate ester (PAHEMA), (**B**) 3-sulfopropyl methacrylate potassium salt (SPM), and (**C**) [2-(methacryloyloxy)ethyl] trimethylammonium chloride (METAC).

Typically, AA, AAm, and their derivatives are widely used in industrial production of SAPs [43,44,45]. Although these hydrogels have excellent swelling properties, the AAm monomer is known for its carcinogenic effects [46,47,48], and AA and its derivatives are associated for causing irritation, allergies, pruritus, and cutaneous eruptions [49,50,51]. Consequently, in human-contact applications, it is imperative to prepare hydrogels with minimal amounts of residual monomers and unwanted byproducts, as hazardous and toxic substances may be generated during synthesis [52]. So, in this study, three monomers unconventionally used in PCPs were evaluated. Figure 2 presents their chemical structures.

These monomers were selected based on their reduced cytotoxicity and potential to improve hydrogel swelling properties through the presence of charge groups. The monomer phosphoric acid 2-hydroxyethyl methacrylate ester (PAHEMA, Figure 2A) has already been studied in hydrogels for biomedical applications [53], scaffold applications [54,55], dental adhesives [56], food contact materials [57], proven its safety and biocompatibility. Additionally, the phosphate group in its structure is expected to improve the biodegradability of the resulting polymer. The 3-sulfopropyl methacrylate potassium salt (SPM, Figure 2B) was used in the synthesis of a SAP with a high swelling of ~2600 g/g [58]. Furthermore, SPM was evaluated as a primary constituent in hydrogels for biomedical applications, exhibiting favorable sterilization properties [59], and anti-biofouling behavior against bacteria [60]. The [2-(methacryloyloxy)ethyl] trimethylammonium chloride (METAC, Figure 2C) was also investigated due to its choline chloride-based structure, as choline is recognized as an essential nutrient with an amino acid-like structure and metabolism [61,62]. In addition to its excellent hydrophilicity [63], the presence of a quaternary ammonium group in its structure can confer and enhance antimicrobial properties in SAPs materials [64,65,66], which is of great benefit for personal hygiene products.

Moreover, the cationic nature of METAC, as opposed to the anionic nature of the other monomers, may result in a hydrogel with ampholytic properties, potentially leading to stronger electrostatic forces and improved absorption properties [67,68].

Before being copolymerized with AC, those monomers were homopolymerized to evaluate their capacity to prepare hydrogels and assess their swelling properties. Homopolymer hydrogels were synthesized according to Table 1, and their maximum swelling properties and gel content were subsequently evaluated.

For all monomers, 1.0% of crosslinker was not enough to prepare structurally stable hydrogels. In the case of the PAHEMA monomer, the homopolymer hydrogel was only achieved with a crosslinking concentration of 5.0%, while other hydrogels were obtainable at concentrations of 3.0%. This observation is remarkable, considering that hydrogels with AAm or AA can be prepared with smaller quantities of crosslinker (less than 0.5% in some instances), although those were prepared by thermal initiation, and for longer periods of time [69,70,71], which immediately suggested potential issues regarding polymerization and crosslinking processes of selected monomers. As expected, hydrogels obtained with increasing amounts of crosslinker presented lower swelling capacity and higher gel content. Also, the maximum swelling capacity does not seem to depend on whether the polymer charge is positive or negative, as evidenced by the values obtained with SPM and METAC, both reaching values above 250 g/g.

All these monomers were also copolymerized with AC to produce hydrogels. Since AC is already a linear molecule with multiple linkage points along its structure, the copolymerization process was expected to increase the gel content and facilitate the incorporation of monomers into the hydrogels. The preparation of hydrogels was carried out stepwise, and several properties were investigated by varying different ratios in the formulations, including the type of monomers, their mass ratios, the crosslink density which was evaluated by using different amounts of crosslinker and initiator, and the mass ratio between AC and monomers. The hydrogels were evaluated based on their maximum swelling properties and their gel content. After these optimization studies, the most promising formulations were selected and fully characterized for the proposed application, alongside a commercial SAP from a baby diaper.

#### 2.2.1. Effect of Monomers Ratio

The incorporation of the selected monomers into cellulose was evaluated considering a 50:50 mass ratio between AC and monomers. In addition, the synergistic effect of the advantages of the different conjugated monomers and their different mass ratios in the formulations were investigated. A cellulose hydrogel (Cel_100_), without added monomers, was also prepared and used as control sample. The formulations are listed in Table 2.

Gelation is the development of a stable three-dimensional network through molecular interactions, whereby a liquid system is transformed into a gel. This process depends on the material and the type of crosslinking, whereby the mechanisms are classified into physical (reversible) and chemical (irreversible) gelation. In the context of acrylate-based monomers with UV initiators, the primary gelation mechanism is free radical polymerization, which falls under chemical gelation. For chemical crosslinking, there are several theoretical models for predicting the gelation process of certain systems. In particular, the Flory-Stockmayer theory predicts gelation thresholds based on the reactivity and functionality of monomer, while the percolation theory describes gelation as a phase transition that occurs when infinite clusters form at a critical crosslink density [72].

Allylic monomers are characterized by their slow polymerization kinetics and the production of low-molecular-weight polymers due to the stability of the propagating radical, which requires higher relative amounts of initiators [73]. However, the cellulose derivative chains with a degree of polymerization (DP) of about 280 offer numerous linkage points due to their functionalization. In this scenario, radical propagation is less critical as it is primarily required to crosslink different cellulose chains rather than to enable extensive chain growth. Cellulose chains can act as both macromonomers and crosslinkers. When additional acrylic monomers are introduced into the formulation, the gelation process depends on the covalent crosslinking of the polymer chains formed and the cellulose backbone. UV light activates the photoinitiator and generates free radicals which also attack acrylate monomers and initiate the chain growth of the polymer. Subsequently, multifunctional products, such as AC or bis-acrylamide allow the polymer chains to crosslink and form an covalent network [74]. It is important to note that cellulose should act as both a macromonomer and a crosslinker in the copolymerization process, where it is expected that the polymer chains will be linked to cellulose via the multiple available coupling sites available. In addition, a bifunctional crosslinker is incorporated to facilitate the covalent linkage of the polymer chains formed in between.

The Cel_100_ hydrogel had a maximum swelling capacity of 8.4 g/g and served as a reference. The incorporation of the monomers proved to be effective in improving the swelling capacity of the cellulose hydrogel, as all formulations tested showed a higher swelling capacity than Cel_100_. This improvement was attributed to the introduction of hydrophilic groups into the polymer matrix, which increased the affinity of the hydrogels for water, as expected. In terms of individual monomers, the incorporation of METAC into cellulose (Cel_50__M_50_) was more effective in the swelling capacity (25.7 g/g) compared to SPM (Cel_50__S_50_, 15.9 g/g) and PAHEMA (Cel_50__P_50_, 13.2 g/g). Contrary to expectations, the combination of two monomers originates gels with lower absorption capacities than individual monomers, even with opposite charges (METAC and SPM/PAEHMA). It is noteworthy that hydrogels combining the three monomers presents the higher overall values of water absorption and the hydrogel Cel_50__P_16.7__S_16.7__M_16.7_, the highest being 29.1 g/g. This could be attributed to a synergistic effect of the three monomers used. The second greatest swelling value was obtained with Cel_50__M_50_ (25.7 g/g), followed by Cel_50__P_12.5__S_25__M_12.5_ (22.2 g/g) (green shaded in Table 2).

Regarding the gel content, hydrogels with higher values correspond to ones with a higher swelling capacity. This suggests an increased degree of incorporation of added monomers in the polymeric crosslinked structure. Considering the results obtained, two formulations (Cel_50__P_16.7__S_16.7__M_16.7_ and Cel_50__P_12.5__S_25__M_12.5_) showed superior swelling results and higher gel contents and were selected for further optimization in terms of crosslink density and the effect the AC/monomers ratios. Also, the presence of the three monomers enabled the investigation of the synergistic effects among the three monomers.

#### 2.2.2. Effect of Crosslinker Quantity

Crosslinking density is a key factor in the swelling behavior of hydrogels. Highly crosslinked polymers tend to have a lower swelling ratio as the polymer structure becomes more compact and limits the expansion of the network [75,76]. Nevertheless, a minimal amount of crosslinker is necessary to link the polymer chains and to obtain a mechanically stable hydrogel, so a balance between the degree of crosslinking and the swelling properties should be investigated [77].

To investigate whether the crosslinking agent added would improve the swelling properties of hydrogels several experiments were carried out by adjusting the crosslinker amount (Table 3). Both formulations (Cel_50__P_16.7__S_16.7__M_16.7_ and Cel_50__P_12.5__S_25__M_12.5_) exhibited similar behavior, which showed that the swelling capacity did not increase significantly when the amount of crosslinker was reduced (from 1.0 to 0.1% *w*/*w* range). The results suggest that lower crosslinker contents cause the network to become mechanically weaker and water is losing due to weakness of the structure. The lower gel content of the hydrogels with lower amounts of crosslinker indicates the formation of non-crosslinked polymer chains that could be released from hydrogel structure. Due to the small difference in the results between 1.0% and 0.5% of crosslinker, the latter amount was chosen for further studies.

#### 2.2.3. Effect of Initiator Amount

The amount of initiator also influences the crosslinking process of hydrogel. To analyze the effects of initiator dosage on the properties of hydrogels, the amount of UV-initiator (2-hydroxy-2-methylpropiophenone) was reduced gradually from 1.0 to 0.25% *w*/*w*, as presented in Table 4. The other optimized hydrogels parameters were kept constant.

The swelling capacity of the hydrogels increased as initiator dosage was reduced, especially when 0.25% was added, resulting in swellings of 60.5 g/g for Cel_50__P_16.7__S_16.7__M_16.7_ and 92.8 g/g for Cel_50__P_12.5__S_25__M_12.5_. However, when comparing the gel contents, the hydrogels showed significantly lower gel contents of 34.0% and 42.5%, respectively, which corresponds to more than a 50% reduction. A decrease in the initiator dosage leads to larger polymer chains between the crosslink points of the network, which promotes more water absorption but less structural integrity. The larger chains that are not crosslinked do not resist water and are released to the medium.

The compromise between gel content and swelling absorption was achieved for formulation with 0.5% of initiator and this amount was used in further optimizations.

#### 2.2.4. AC: Monomers Ratio Influence

The relationship between the mass ratio of AC and monomers used in the preparation of hydrogels and the water absorption capacity was investigated, by varying the mass ratio between 50:50 and 10:90 range (Table 5). All other parameters were set according to the previously optimized values.

The maximum absorption capacities of 102.3 g/g for Cel_10__P_22.5__S_45__M_22.5_ and 94.6 g/g for Ce1_10__P_30__S_30__M_30_ were achieved with formulations with lower amount of cellulose (10% *w*/*w*). This phenomenon is due to an increase in the polymer contents which introduces more hydrophilic groups into the polymer network, that by solvation can attract and retain more water molecules. Curiously, in hydrogels with 25% of cellulose this effect was not observed, which suggests that cellulose acts as a strong backbone of the hydrogel network, strongly affecting the hydrogel swelling. In some ways, the cellulose backbone structurally limits the water absorption, and these limits are overpassed when the cellulose amount decreases to 10%. Nevertheless, the decrease in AC content is likely to affect and hinder the biodegradability of the hydrogels, as the cellulose backbone is expected to be more susceptible to biodegradation than the other polymers formed [24,43,78].

Overall, it was confirmed that several parameters play an important role in the structure of the hydrogels and consequently influence water uptake and gel fraction. Particularly, the Cel_50__P_16.7__S_16.7__M_16.7_ and Cel_50__P_12.5__S_25__M_12.5_ initially had maximum swellings of 29.1 g/g and 22.2 g/g, respectively, while maximum absorbance values of 94.6 g/g and 102.3 g/g were achieved after optimization of the parameters.

It should be emphasized that the formulations obtained were optimized with respect to the tested concentrations; however, for practical applications, further tests should be conducted to determine the precise optimal values.

### 2.3. Evaluation of the Most Promising Cellulose-Based Hydrogels for Practical Applications

Based on the above findings, formulations with PAHEMA:SPM:METAC monomers in a 1:2:1 mass ratio were selected for further studies. This decision relied on the observation that formulations with this ratio of monomers consistently originated hydrogels with higher water absorption capacity and higher gel contents, comparatively to 1:1:1 monomers mass ratio, suggesting that SPM is more easily converted and consequently incorporated into the hydrogel structure [79,80]. From the results obtained, and considering the maximum swelling capacity, three hydrogel preparations based on a 1:2:1 mass ratio of monomers were selected for further investigations (Table 6), evaluating practical applications.

The main objective of the selected formulations was to evaluate the highest swelling values obtained so far, considering the effect of the AC/monomers ratio, and the initiator amount added. As control samples, the hydrogel of only cellulose (Cel_100_) and the isolated SAP from a commercially available baby diaper (DiaperA) were also fully characterized.

#### 2.3.1. FTIR Spectroscopy of Hydrogels

**Figure 3:**
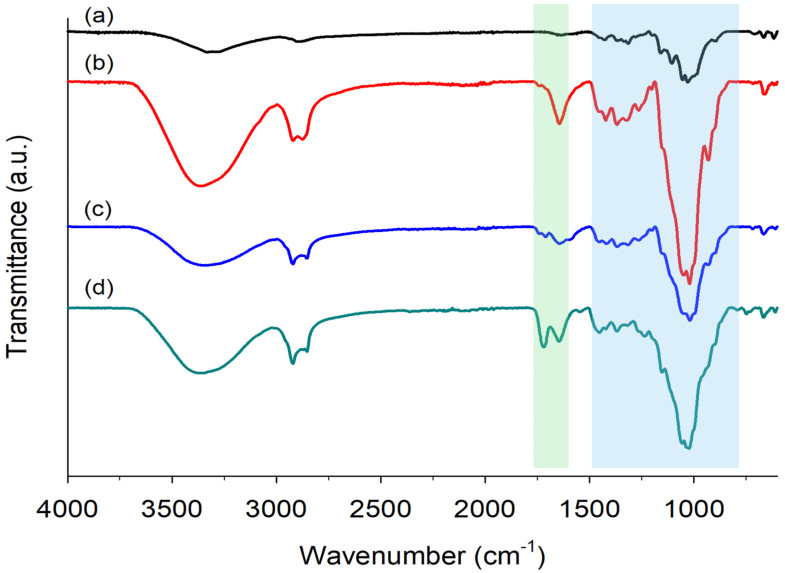
FTIR spectra of dried samples of (a) cellulose powder, (b) AC derivative, and of hydrogels (c) Cel_100_, and (d) Cel_50__P_12.5__S_25__M_12.5_.

The FTIR spectra (Figure 3) of dried hydrogels samples were acquired to confirm the AC (co)polymerization and monomers incorporation into hydrogels. For the Cel_100_ hydrogel (Figure 3c), the cellulose backbone peaks were detected, as expected, and the observed reduction in the signal at 1650 cm^−1^ (C=C) indicates the disappearance of this group in the crosslinking of cellulose chains [81]. As FTIR spectroscopy is typically used as a qualitative technique, the only expected difference in the spectra of Cel_50__P_12.5__S_25__M_12.5_ and Cel_50__P_16.7__S_16.7__M_16.7_ is the relative intensity of the bands with respect to the SPM monomer. Concerning the copolymerization of AC with the other monomers, it was quickly noticed that the bands of the polymers formed were masked by the bands of the cellulose backbone, so the characteristic peaks between 1500–830 cm^−1^ of the corresponding copolymers are masked by the characteristic peaks of the cellulose backbone. Consequently, the typical peaks associated with PAHEMA (ν P=O at 1093 cm^−1^ and ν P-O-C at 985 cm^−1^) [82,83], SPM (ν_s_ at 1172 cm^−1^ and ν_as_ at 1043 cm^−1^ of SO_3_ group) [84], and METAC (ν C-N of quaternary ammonium group at 956 cm^−1^ and δ C-N of ammonium methyl group at 1474 cm^−1^) [85] were not possible to detect in the Cel_50__P_12.5__S_25__M_12.5_ hydrogel spectrum. The analysis of Cel_50__P_12.5__S_25__M_12.5_ spectrum (Figure 3d), revealed the appearance of peaks at 1726 cm^−1^ and 1646 cm^−1^, which were attributed to the carbonyl group (ν C=O) of the copolymers [84,85,86]. Furthermore, the FTIR spectra were only acquired in this sample because this mass ratio of monomers was selected as the most promising hydrogels for practical applications.

#### 2.3.2. Thermal Properties

**Figure 4:**
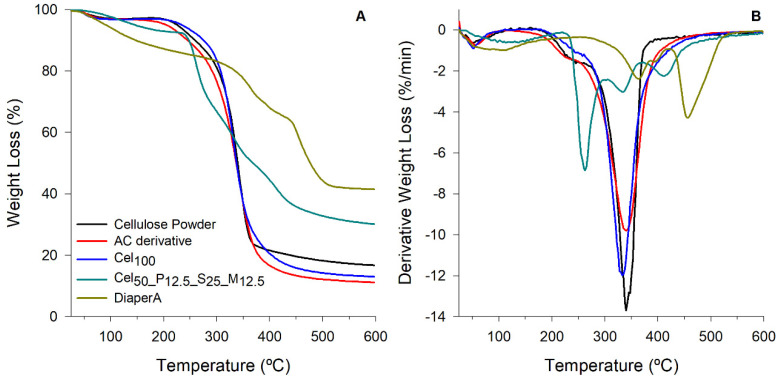
(**A**) Weight loss curves of cellulose powder, AC derivative, Cel_100_, P_100__c_5_, M_100__c_5_, Cel_50__P_12.5__S_25__M_12.5_, and DiaperA; (**B**) respective derivatives of weight loss with respect to time (dTG).

Thermogravimetric analysis (TGA) and derivative thermogravimetry (dTG) curves elucidate the thermal degradation behavior of the materials (Figure 4). Table S1 presents a summary of the thermal degradation temperatures of the prepared samples and the isolated diaper absorbent (DiaperA).

All thermograms demonstrated a small weight loss up to 110 °C due to evaporation of adsorbed water. In cellulose powder thermogram, a mass loss was observed in the range of 250–350 °C, corresponding to the typical dehydration and decomposition of the cellulose. AC exhibited a lower thermal stability than cellulose powder, with a small shoulder observed around 240 °C (more evident in dTG graph) attributed to the degradation of the allyl pendant moieties [38,87]. After polymerization, Cel_100_ samples exhibited an increased thermal stability, suggesting a more compact and stable structure, as expected.

The Cel_50__P_12.5__S_25__M_12.5_ sample demonstrated significantly higher thermal stability up to 250 °C, compared to DiaperA, with a 5% loss occurring only at 145.9 °C (Table S1). The dTG peak at 262.1 °C was identified as the removal of quaternary ammonium groups of METAC [88] and the degradation of sulfonyl groups [89], and the peak at 334.3 °C to cellulose decomposition [90,91]. Furthermore, the third stage identified by dTG peak at 410.2 °C was attributed to the decomposition of METAC [88], PAHEMA [92], and SPM [93] backbones itself.

The DiaperA sample exhibited the lowest thermal stability up to 250 °C. Although the diaper absorbent main degradation step occurs at a higher temperature (around 500 °C), it has lower degradation temperatures (T_95_ and T_90_), which indicates an earlier onset of degradation, that could be attributed to the volatilization of unreacted monomers, such as typically used AA and AAm, whose boiling points typically range between 120 and 141 °C. Additionally, the DiaperA sample showed the highest residue levels of 41.5%. This high residual level is typically associated with the presence of inorganic parts, probably due to the counter-ions present, as in potassium polyacrylate. Although our developed hydrogels also possess some residual level, higher than the all-cellulose samples, its content was lower than in the commercial sample. While most commonly used synthetic monomers offer a main degradation step at a higher temperature, the high residue levels indicate a substantial presence of inorganic or non-combustible components, which pose environmental challenges, particularly in waste management and/or recycling [8,9].

#### 2.3.3. Swelling Capacity

Swelling tests were conducted to assess the adsorption capacity and swelling kinetics of the chosen samples. For practical applications, hydrogels are typically employed in powder form; therefore, a particle-size analysis was initially performed. Then, additional parameters were evaluated, including AC/monomers ratios, the influence of crosslinking density and gel content, and the effects of fluid salinity and pH on swelling capacity.

Particle-size and morphology impact on the swelling capacity

Previous evaluations of the properties of hydrogels were carried out with circular-shaped samples (Ø = 20 mm). However, for practical applications, they are applied in a powder form and so three different methods were used to convert the dried hydrogel samples into a powder form. Dried hydrogels were subjected to grinding under three distinct conditions: (1) cryogenic fracture using a mortar and pestle (CF), (2) ground in a coffee grinder (CG), and (3) coarse manual broken (MB) (Figure S4).

**Figure 5:**
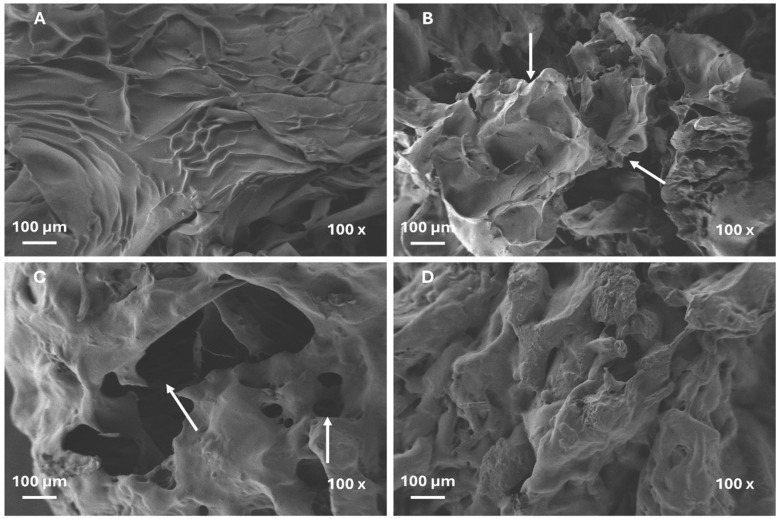
SEM images of surfaces of Cel_10__P_22.5__S_45__M_22.5_ hydrogel prepared under different grinding conditions: (**A**) circular disks of 20 mm; (**B**) ground in a coffee grinder (CG); (**C**) cryogenic fracture (CF), and (**D**) manually broken (MB).

Scanning electron microscopy (SEM) images of the surface of Cel_10__P_22.5__S_45__M_22.5_ ground hydrogels samples prepared through different methods are presented in Figure 5. The surfaces of the original disk samples (Figure 5A) maintained a rough and intact surface morphology. Both coffee grinder (Figure 5B) and cryogenically fractured (Figure 5C) methods clearly revealed surface damage of samples, with sharp edges, fragmented and larger fissures (arrows in figure). These processes are likely due to considerable shear forces, which are generated during grinding that causes extensive fragmentation of the samples’ surface. In contrast, disk samples and manually broken hydrogels (Figure 5D) showed a more continuous, compact, and less-disrupted structure.

**Figure 6:**
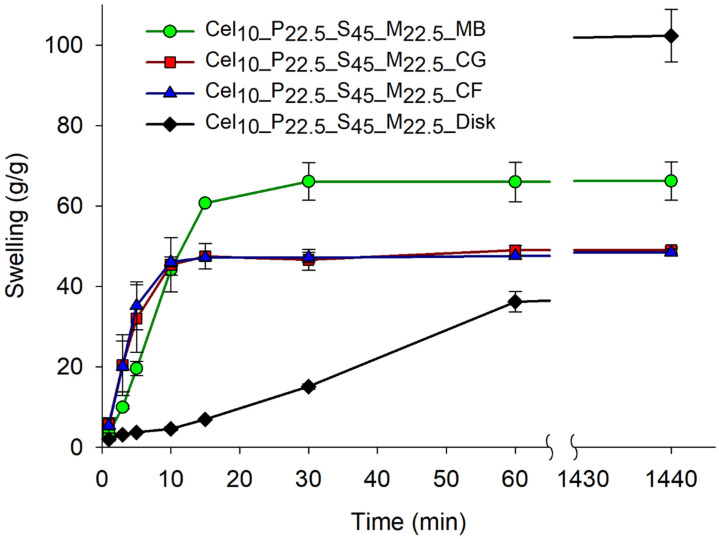
Grinding method evaluation in swelling properties of Cel_10__P_22.5__S_45__M_22.5_ hydrogel. Three preparation methods were compared: cryogenic fracture (CF), coffee grinder (CG), and coarsely manually broken (MB). The swelling ratio of disk samples (20 mm) was also evaluated. Swelling was measured at different time intervals up to equilibrium (after 24 h).

Also, the swelling kinetics of the Cel_10__P_22.5__S_45__M_22.5_ hydrogel samples subjected to the three different griding processes were analyzed to evaluate how this process affects water absorption (Figure 6). In the initial 10 min, all three methods exhibited fast and comparable swelling kinetics. Subsequently, distinct differences in their performance became apparent. The CF and CG methods showed similar swelling behavior, with equilibrium swelling ratios of about 48 g/g, whereas the MB hydrogel exhibited superior performance, attaining ~67 g/g. However, none of the ground methods approached the maximum value previously obtained (102.3 g/g) with disk samples. Nevertheless, after 15 min, all ground samples reached their maximum swelling capacity, demonstrating rapid swelling kinetics, which were not observed in disk samples (black line). A gap was made in the time axis to a better understanding and larger representation of the initial time points. In Figure 6, the black line refers to the disk sample which showed slower swelling kinetics. While the other ground samples had already reached their maximum swelling after 60 min, and the graph line therefore shows the same value, the disk sample was still increasing its absorbency, resulting in the observed cut in the graph. In PCPs, rapid absorption is crucial to efficiently trapping liquids, preventing leakage and maintaining skin dryness. In this regard, the smaller particles, with a larger surface area-to-volume ratio, facilitated faster water absorption, enabling the material to reach equilibrium quickly. Thus, grinding methods that produce smaller particles proved highly relevant for personal care product applications, but the results also suggest that certain ground methods may compromise the structure of cellulose-based hydrogels, significantly impacting swelling performance.

The SEM images revealed a direct correlation between the hydrogels’ morphological structure and their absorption capacity. The introduction of significant structural disruptions of polymer networks resulted in a substantial reduction in water uptake. Conversely, non-destructive methods are likely to preserve the structure and result in maximal retention and absorption; however, these methods led to slower absorption kinetics.

Manual fragmentation, although it induces moderate structural disruption, was selected as the preparation method for further characterizations because it presented the better balance between rapid swelling kinetics and maximum absorption capacity.

Fluids salinity influence

**Figure 7:**
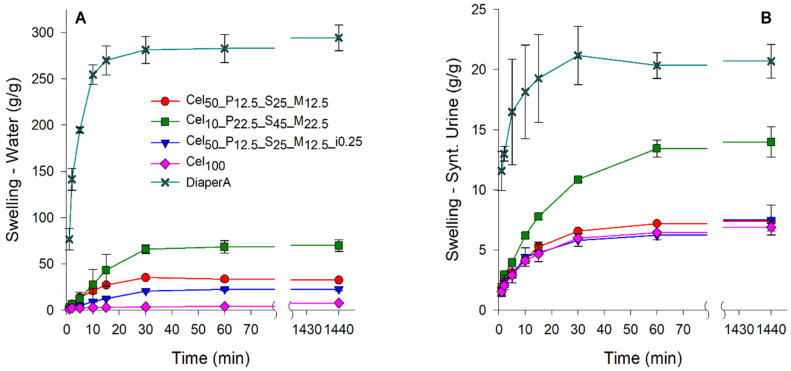
Influence of fluids salinity in swelling capacity of tested hydrogels. (**A**) Free swelling capacity in deionized water. (**B**) Free swelling capacity in synthetic urine.

Fluid salinity is one of the factors influencing the swelling capacity of hydrogels. This factor was investigated, as the proposed application requires salt-resistant hydrogels to ensure high swelling in the presence of saline solutions [94,95]. The swelling kinetics of selected hydrogels are present in deionized water (Figure 7A) and in synthetic urine (Figure 7B). In deionized water, the differences in absorption between the prepared hydrogels and the SAP from diapers were particularly clear. DiaperA SAP showed a significantly higher water absorbing capacity, attaining a maximum absorption of ~295 g/g. Among the prepared hydrogels, the Cel_10__P_22.5__S_45__M_22.5_ formulation proved to be the most remarkable, as it achieved a swelling ratio of 69 g/g. The remaining hydrogels exhibited significantly lower swelling capacities, ranging from 5 to 10 g/g, which was particularly unexpected, particularly for the Cel_50__P_12.5__S_25__M_12.5__i0.25 formulation which had previously achieved a swelling capacity of ~93 g/g in disk samples. This result is possibly due to the lack of a compact structure and low gel content, resulting in a significant reduction in swelling after grinding. Despite the higher swelling capacity of DiaperA in water, the introduction of salinity into the medium led to a drastic decrease in its maximum swelling capacity. Although the highest swelling capacity of DiaperA was maintained in synthetic urine, the ability of hydrogels to absorb liquid solution was significantly reduced by the presence of salts, resulting in a decrease in the maximum swelling from ~295 g/g to ~20 g/g. Regarding the prepared cellulose-based samples, Cel_10__P_22.5__S_45__M_22.5_ showed the highest swelling capacity (~15 g/g), which narrowed the gap to DiaperA, compared to the results obtained in water. The other cellulose-based hydrogel formulations had maximum swelling values between 6.9 and 7.5 g/g and showed no substantial difference between water and synthetic urine.

Fluids pH effect

**Figure 8:**
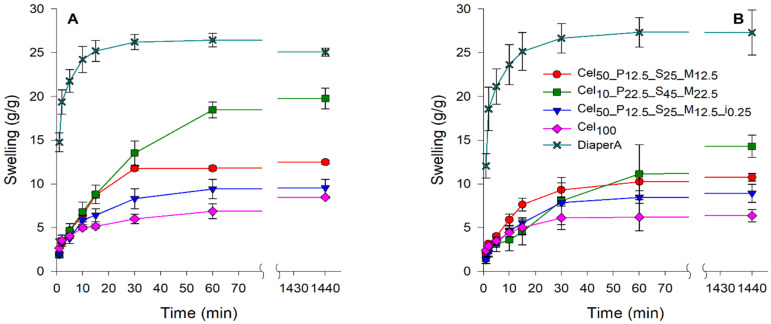
Influence of pH in swelling capacity of tested hydrogels. (**A**) pH 4 buffer; (**B**) pH 8 buffer.

Ionic superabsorbent hydrogels show different swelling behavior at different pH ranges. The swelling capacity of the developed hydrogels was tested in buffer solutions with pH values of 4 and 8 (Figure 8), as these pH values correspond to the physiological pH extremes of human urine, which is normally slightly acidic (5.5 to 6.5) [96]. The samples tested generally showed higher absorption in buffer solutions than in synthetic urine, probably due to the lower amounts of salts concentration and protonation/deprotonation of the functional groups present in the hydrogels. At pH 4, the DiaperA sample exhibited a swelling of ~25 g/g, while Cel_10__P_22.5__S_45__M_22.5_ performed remarkably well at ~20 g/g, which was the closest gap to DiaperA compared to all other media tested. DiaperA performed similarly at pH 8 as at pH 4; however, at pH of 8 the Cel_10__P_22.5__S_45__M_22.5_ hydrogel absorbed ~15 g/g, which was lower than in the acidic medium. It was expected that the presence of anionic and cationic groups in the developed hydrogels could balance the sensitivity of swelling to pH, as the swelling properties of hydrogels are determined by the ionization degree of their functional groups [97].

Although the pH tested were acidic or basic, sulfonic acid is a very strong acid (pKa ~ −7), so sulfonic groups (from SPM) are expected to be in their charged form (sulfonate anions), being their influence in swelling at different pHs tend to be zero [98]. Also, the quaternary ammonium cationic group of METAC cannot be protonated/deprotonated, so its influence at different pHs is also expected to be negligible [99]. PAHEMA being an ester of phosphoric acid is the most affected to changes in pH medium. At pH 4, the acid group of PAHEMA is partially protonated, so that the electrostatic repulsion between negatively charged groups tends to decrease, which can lead to a lower swelling capacity. However, at basic pH, the opposite occurs, and the phosphate group becomes ionized, resulting in higher electrostatic repulsion. Nevertheless, this effect on swelling properties of hydrogels was not observed. This could be because the appearance of negative charges of PAHEMA can neutralize the positive charges that already exist (from METAC), which can lead to the displacing of the counter-ions present in the hydrogel structure, and consequently, to a lower swelling capacity. Also, the increase in cations (from the negatively charge groups from—PAHEMA and SPM) can exert a screening effect over the negative charged groups. Due to this, the repulsive forces between the negative polymer chains are reduced, thus reducing the total swelling capacity [58,100]. Additionally, the use of buffer solutions can indirectly influence the swelling capacity of hydrogels through the addition of ions to the swelling medium [101].

Among the hydrogels prepared, Cel_10__P_22.5__S_45__M_22.5_ showed consistently better swelling ratios compared to the other formulations under all conditions. The results obtained indicate that, although the cellulose-based hydrogels could not match the performance of commercial SAP in deionized water, they exhibited promising swelling capacities in synthetic urine and pH buffers, especially in the acidic medium, suggesting their potential for applications in PCPs. Moreover, the swelling of the hydrogels prepared in this study exceeded the swelling capacities of some other cellulose-based superabsorbents in water (15–80 g/g) [102,103] and in synthetic urine/saline solutions (5–16 g/g) [104,105]. Also, Cel_10__P_22.5__S_45__M_22.5_ achieved higher swelling values in synthetic urine than some studied commercial diapers absorbents (9–15 g/g) [106].

#### 2.3.4. Fluids Retention Capacity of Hydrogels

**Figure 9:**
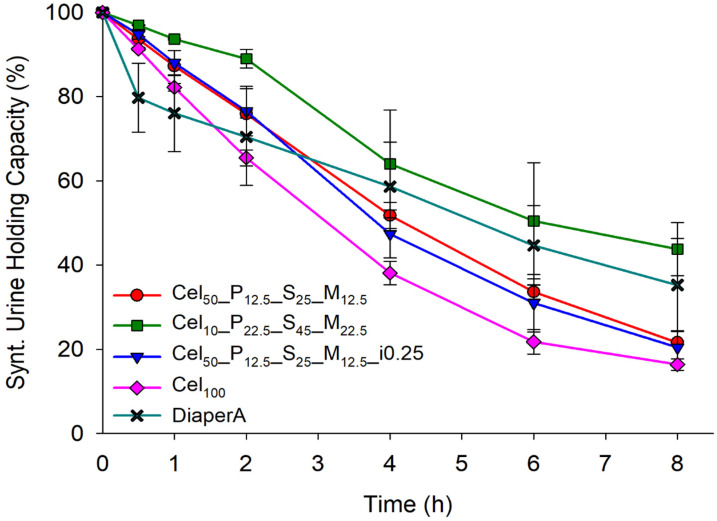
Synthetic urine retention capacity of tested hydrogels samples over an 8 h period.

In addition to the requirements for rapid and high liquid absorption for PCPs, SAPs should also have excellent liquid retention, a property not often evaluated by evaporation. The presence of salts in solutions can interfere with the ability of polymers to absorb and retain liquids. The retention capacity of synthetic urine was evaluated over a period of 8 h (Figure 9). DiaperA showed a significant decrease in retention to 70% within the first 2 h, while the prepared cellulose-hydrogels showed greater capacity in retaining synthetic urine. Comparing all the samples, DiaperA showed higher retention of synthetic urine than most cellulose hydrogels, but less than the Cel_10__P_22.5__S_45__M2_2.5_ hydrogel. In the full-time experiment, Cel_10__P_22.5__S_45__M_22.5_ showed the highest performance by retaining about 44% after 8 h, while DiaperA retained only 35%, indicating its potential for applications requiring higher liquid retention in PCPs.

#### 2.3.5. Centrifuge Retention Capacity (CRC) of Hydrogels

**Figure 10:**
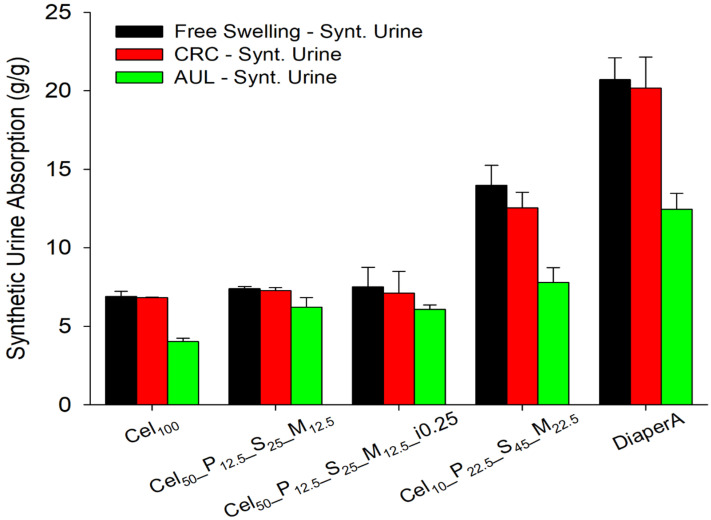
Synthetic urine centrifuge retention capacity (CRC) (**middle** bars) and absorbency under load (AUL) (**right** bars) capacities were measured in the chosen samples and controls (Cel_100_, and DiaperA). The equilibrium swelling in synthetic urine (**left** bars) is presented as a reference.

The CRC method was used to determine the retention of synthetic urine in hydrogels after swelling and centrifugation under standardized conditions (Figure 10). In previous studies, it was found that water in a hydrogel can be divided into three types: bound water, half-bound water, and free water.

In a swollen SAP matrix, free water exhibits greater mobility compared to bound and half-bound water, making it more susceptible to loss [107]. Furthermore, the proportions of bound and half-bound water in the swollen hydrogels correlate directly with the concentration of hydrophilic groups present in a given volume of superabsorbent material [101]. All samples exhibited favorable CRC and reached final retention values approaching the maximum swelling capacities, with CRC values exceeding 90%. Comparing the CRC values with the FSC results, the stability of these hydrogels under centrifugation is evident, as the hydrogels were able to retain a high percentage of the absorbed liquid, indicating mechanical stability at full saturation.

#### 2.3.6. Absorbency Underload (AUL) of Hydrogels

The AUL method is a standard test often used for evaluation of disposable baby diapers SAPs, which evaluate the swelling capacity of hydrogels under a specific applied load to the sample (50 g·cm^−2^). This test is critical as it evaluates the ability of absorbent materials to maintain their swollen strength when subjected to compression during the swelling process. In addition, the applied load can significantly affect the structure of the material, leading to morphological changes and even the collapse of its internal configuration [108].

As expected, the value of the AUL test (Figure 10) was significantly lower than those of the FSC, for all tested samples. A trend was observed except for the Cel100 sample. The higher the cellulose content in the formulation, the lower the AUL loss swelling capacity. For the Cel_100_, Cel_10__P_22.5__S_45__M_22.5_, and DiaperA samples, the decrease in AUL capacity was about 50% of the FSC, indicating a lack of mechanical strength and structural integrity that does not support the applied load. Remarkably, the loss of swelling capacity in AUL test at a 50:50 ratio of cellulose to monomers was only about 20%, confirming previous results that cellulose acts as the backbone of the hydrogel network, and improves mechanical performance due to a higher crosslinking density and the better mechanical properties of cellulose [43,109].

It is clear that a balance between swelling capacity and mechanical performance should be achieved regarding the final application, as these properties are inversely related, and the hydrogels with the highest swelling capacity showed the lowest capacity under load conditions. Nevertheless, the developed cellulose-hydrogels outperformed the AUL values of other cellulose-based absorbents for feminine hygiene applications [104].

#### 2.3.7. Cytotoxicity Evaluation of Hydrogels

**Figure 11:**
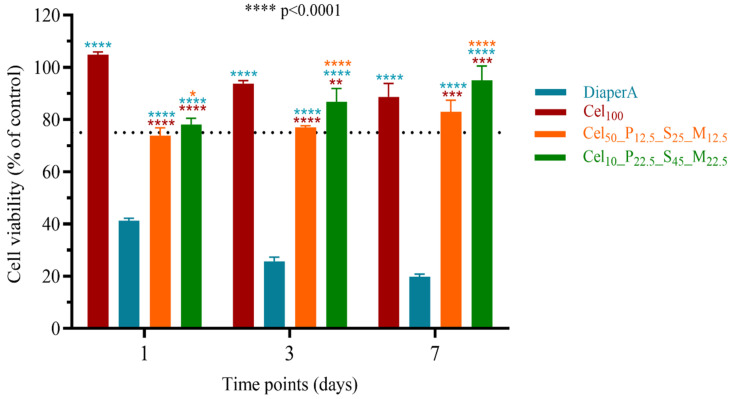
Cell viability results of hydrogels in direct contact with NHDF cells. Data represents the mean ± SD, n = 3. The * indicates significant difference (**** *p* < 0.0001, *** *p* = 0.0008, ** *p* = 0.0023 and * *p* = 0.0134) between samples on the same day of culture.

The cytotoxicity of the developed hydrogels was determined by quantitative analysis with the alamarBlue™ HS Cell Viability Reagent [110]. For this purpose, NHDF fibroblast cells were cultured on the surface of the hydrogels for 1, 3 and 7 days. Untreated cells (without hydrogels) were used as controls. The results are shown in Figure 11 in percentage of cell viability (%). According to ISO 10993, materials with cell viability greater than 75% are considered non-cytotoxic [111].

The cytotoxicity test showed that the DiaperA hydrogels had a cytotoxic effect on NHDF cells with a cell viability below 75% at all time intervals tested. Furthermore, hydrogels containing only cellulose (Cel_100_) showed a significantly greater percentage reduction in alamarBlue, indicating a favorable cellular response, as expected.

Compared to untreated cells, the cell viability of Cel_10__P_22.5__S_45__M_22.5_ hydrogels was 78.20 ± 2.30% for 1 day, 86.84 ± 5.07% for 3 days and 95.043 ± 5.54% for 7 days, indicating that the NHDF cells maintained their metabolic activity, i.e., the Cel_10__P_22.5__S_45__M_22.5_ hydrogels were not cytotoxic to cultured cells. A similar behavior was observed with the Cel_50__P_12.5__S_25__M_12.5_ hydrogels, although the viability after 1 day was slightly lower than 75% (73.89 ± 2.95%), the results showed a viability above 75% at 3 (77.07 ± 0.59%) and 7 (82.90 ± 4.58%) days, indicating a favorable environment to cells proliferation [112].

It was expected that a higher percentage of cellulose would have a lower cytotoxicity effect; nevertheless, the results obtained for Cel_50__P_12.5__S_25__M_12.5_ and Cel_10__P_22.5__S_45__M_22.5_ showed the contrary, confirming that the use of the selected monomers copolymerized with cellulose was correct and their presence does not affect cells’ viability. One important aspect of cell viability during time is that the absorbent of DiaperA causes a constant decrease instead of the prepared cellulose-based hydrogels that showed a constant increase in cell viability. The cellulose-based formulations showed higher cytocompatibility than the SAP from diaper, proving that commercial SAPs should be continuously investigated, improved, and replaced by more sustainable and biocompatible materials.

## 3. Conclusions

In this study, unconventional cellulose-based hydrogels were successfully developed as a sustainable alternative to conventional synthetic superabsorbent polymers commonly used in personal care products. The research focused on the synthesis of an AC derivative and its copolymerization with unusual monomers used in SAPs, including PAHEMA, SPM, and METAC, through the FRP process, a simple, affordable, and easy to implement method, commonly used in the industry. By optimizing formulation parameters such as monomers ratio, crosslinking density, and AC/monomers mass ratio, the cellulose-based hydrogels were able to significantly improve their swelling capacity, from 22 to 102 g/g.

Among the formulations tested, the Cel_10__P_22.5__S_45__M_22.5_ hydrogel formulation demonstrated promising properties for personal care applications, particularly in absorbent products. This cellulose-based hydrogel exhibited an absorption capacity of 15 g/g in synthetic urine and 20 g/g in acidic medium, approaching the performance of commercial SAPs used in baby diapers. The retention capabilities of these cellulose-based hydrogels were also noteworthy, maintaining 45% of their absorbed fluid after 8 h. This retention performance suggests potential benefits for extended-use personal care products, where maintaining dryness over time is crucial. A significant advantage of these developed hydrogels lies in their superior biocompatibility. Cell viability tests revealed that these hydrogels provided a cell viability above 75%, indicating minimal cytotoxic effects, increasing the cell viability over time. In contrast, commercially available SAPs exhibited considerable cytotoxicity, with a maximum cell viability of only 42% after 1 day, being reduced over time. The combination of adequate absorption capacity, comparable retention capabilities, and significantly enhanced biocompatibility potential these cellulose-based hydrogels as promising alternatives to current synthetic SAPs in personal care products. Furthermore, the use of cellulose-based materials aligns with growing trends towards sustainable and biodegradable products in the personal care industry. This eco-friendly aspect, coupled with the improved safety profile, could provide a competitive edge in the market.

Although further optimization is required to achieve the maximum swelling capacity of commercial SAPs in deionized water, these cellulose-based hydrogels show significant potential as more sustainable and biocompatible alternatives for hygiene products. Their improved retention properties, pH sensitivity, and reduced cytotoxicity could transform the hygiene products industry by offering a better balance between performance, safety, and environmental sustainability. Future research should focus on improving the absorption capacity, particularly under load, while maintaining the favorable biocompatibility of these materials.

## 4. Materials and Methods

### 4.1. Materials

Industrial eucalyptus cellulose dissolving pulp (EDP, DP≃1175) was kindly supplied by Biotek pulping mill (Altri, SGPS, SA, V. V. de Rodão, Portugal) and used without further purification. Sodium hydroxide (NaOH, 99%) was bought from JGMS (Odivelas, Portugal), and Urea ((NH_2_)_2_CO, 99%) from ChemLab (Zedelgem, Belgium). Allyl glycidyl ether (AGE, 99+%), 2-hydroxy-2-methylpropiophenone (96+%), and 2-(methacryloyloxy)ethyl trimethylammonium chloride (METAC, 80% in water) were acquired from TCI (Zwijndrecht, Belgium). Phosphoric acid 2-hydroxyethyl methacrylate ester (PAHEMA, 90%) and 3-sulfopropyl methacrylate (SPM, 98%), were obtained from Sigma-Aldrich (Hamburg, Germany). N,N’-methylenebisacrylamide (BAAm, 98%) was sourced from Riedel-de Haën (Seelze, Germany). Normal Human Dermal Fibroblasts (NHDF, Sigma-Aldrich, Second St. St. Louis, MO, USA) were cultured in the following medium: Minimal Essential Medium (MEM, HyClone™, Cytiva, Freiburg, Germany) supplemented with 10% (*w*/*v*) heat-inactivated fetal bovine serum (FBS, Biowest, Nuaillé, France) and 1% (*w*/*v*) penicillin-streptomycin (Biowest). Deuterium oxide (99.90% D) was acquired from Eurisotop (CotecNet, Ulis, France). Commercial diapers were purchased from Mercadona^®^ (Coimbra, Portugal). Deionized water was obtained through reverse osmosis. All reagents were utilized as received.

### 4.2. Methods

#### 4.2.1. Cellulose Pulp Pre-Treatment

The cellulose pulp was subjected to sulfuric acid hydrolysis to decrease the degree of polymerization (DP) of the cellulose chains, as reported in our previous studies [38,39,109]. Specifically, a predetermined cellulose pulp sample (150 g) was initially mechanically defibrillated using a coffee grinder until a cotton-like material was obtained. Subsequently, the ground pulp was hydrolyzed with sulfuric acid (10.5 mL) and mechanically stirred for 36 h at 450 rpm at room temperature. The resultant cellulose powder was vacuum filtered, added to a 0.5% NaOH/ethanol solution for neutralization, washed twice with ethanol, and dried in an oven at 50 °C prior to use. The final DP was estimated using the intrinsic viscosity method (ISO standard 5351) [113,114].

#### 4.2.2. Allyl Cellulose (AC) Derivative Preparation

A predetermined amount of cellulose powder (6.0 g) was gradually added into a 7/12/81 wt.% of NaOH/urea/water precooled solution (~5 °C) and stirred until a homogeneous suspension was achieved. Subsequently, the suspension was frozen overnight, at −18.0 °C, and then thawed under vigorous magnetic stirring to obtain a yellowish transparent cellulose solution [115,116]. The cellulose solution was then transferred to a three-neck round bottom flask equipped with a dropping funnel, and the system was sealed and purged with nitrogen for 15 min. Allyl glycidyl ether (AGE) in a 5:1 molar ratio of AGE:AGU was added dropwise to the cellulose solution. The reaction proceeded for 24 h at 30 °C under a nitrogen atmosphere [35,38,117]. The reaction solution was washed with cold acetone to remove the excess AGE, and then 50 mL of distilled water was added to redissolve the cellulose crude. The cellulose solution was then dialyzed (MWCO = 3500 Da) against deionized water until a neutral pH was attained. Then, AC was obtained by freeze-drying and stored in a freezer protected from light.

#### 4.2.3. Cellulose Hydrogels Preparation

Cellulose-based hydrogels were synthesized through the following procedure: a predetermined amount of AC (depending on AC/monomers ratio in formulation) was dissolved in 10 mL of deionized water and stirred until complete dissolution. Subsequently, the predetermined quantity of monomers and crosslinking agent (BAAm) (considering the mass ratios in formulations) were incorporated into the solution. Upon achieving a homogeneous solution, the predetermined amount of UV-light initiator (2-hydroxy-2-methylpropiophenone) was added. Typically, the solution was prepared for a 10% mass concentration of AC/monomers. The resultant solution was then transferred to a Petri dish and exposed to UV radiation for 2.5 h. Following polymerization, the resultant hydrogels underwent washing, solvent exchange with ethanol, and air-dried at room temperature, until constant weight [118]. For hydrogels formulation optimization purposes, samples were cut into circular shapes (Ø = 20 mm). To evaluate some practical properties, the hydrogels were ground using three distinct methods: (1) by cryogenic fracture, (2) using a coffee grinder, and (3) coarse manual fragmentation. Three different monomers were evaluated for the preparation of cellulose-based hydrogels. Monomers mass ratios, amounts of crosslinking agent and initiator, and AC/monomers mass ratio were investigated and optimized (Section 2.2).

#### 4.2.4. Isolation of SAP from Commercial Diaper

**Figure 12:**
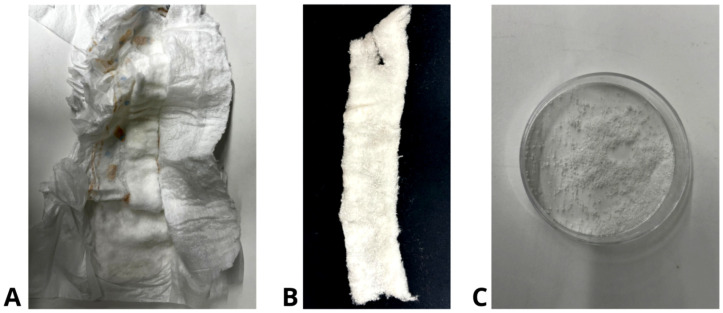
Process to isolate SAP from commercial baby diaper. (**A**) Open core of a baby diaper; (**B**) absorbent core of the diaper; (**C**) SAP sample extracted and isolated by sieving.

The absorbent core of a commercial baby diaper (DiaperA) was accessed by meticulously incising along the sides with a scalpel (Figure 12A). Subsequently, the absorbent core was extracted (Figure 12B), and the superabsorbent polymer was isolated through sieving (Figure 12C).

### 4.3. Characterizations

#### 4.3.1. Fourier-Transform Infrared Spectroscopy (FTIR)

FTIR spectra were acquired utilizing a Nicolet™ iS20 Spectrometer (ThermoFisher Scientific, Brno, Czech Republic) equipped with a Smart iTX—Diamond ATR accessory. The analysis was conducted with a resolution of 4 cm^−1^ and 64 accumulations. The spectra were obtained within the range of 4000–600 cm^−1^, at room temperature, and subsequently subjected to analysis using Curve Manager software 6.00 (ACD/Labs, Canada), version 6.00.

#### 4.3.2. Nuclear Magnetic Resonance Spectroscopy (NMR)

Nuclear magnetic resonance (NMR) spectra were acquired using an Avance III HD 400 MHz spectrometer (Bruker, Ettlingen, Germany) at a temperature of 25 °C. The instrument was equipped with a 5 mm TIX triple resonance detection probe.

#### 4.3.3. Morphology

Hydrogels samples were prepared, then lyophilized. Gold coated hydrogels surface samples were then observed using a field emission scanning electron microscope (SEM), ZEISS MERLIN (Compact/VPCompact, Gemini II, Oberkochen, Germany), with an accelerating voltage of 1.0 kV.

#### 4.3.4. Thermal Studies

Thermogravimetric analysis (TGA) was used to investigate the thermal stability of the hydrogels. A NETZSCH TG 209F1 Libra (Netzsch, Selb, Germany) instrument was used, operating at a heating rate of 10 °C·min^−1^ within a temperature range of 25 °C to 600 °C, under a nitrogen purge flow of 250 mL·min^−1^.

#### 4.3.5. Gel Fraction

The gel fraction (GF) is a quantitative measure that determines the stability and efficiency of crosslinked hydrogels. Specimens of prepared hydrogel (disks Ø = 20 mm) were initially dried in an oven at 40 °C and weighed (W_i_); subsequently, they were immersed in distilled water for 48 h at room temperature to remove any soluble/uncrosslinked materials. The disk samples of hydrogels were removed and dried again until a constant weight was achieved (W_f_). The GF was calculated as [119]:

GF (%) = W_f_/W_i_ × 100 (1)

#### 4.3.6. Free-Swelling Capacity

Free-swelling capacity (FSC) was evaluated by gravimetric method. The prepared dried hydrogels samples were weighed (W_dry_). These samples were subsequently immersed in deionized water and weighed at predetermined time intervals (W_t_) to evaluate the swelling behavior. Equilibrium swelling measurements were obtained at 24 h. All measurements were conducted at room temperature. Excess surface water was meticulously removed with absorbent paper to ensure accurate mass determination. The degree of swelling was calculated based on the ratio between the mass of the swollen gel and the dry gel, using the following formula [120]:

FSC (g/g) = (W_t_ − W_dry_)/W_dry_ (2)

#### 4.3.7. Swelling—Synthetic Urine and pH Buffers

Hydrogels were subjected to swelling tests in synthetic urine and buffer solutions at pH 4 and pH 8. The swelling ratio was calculated using Equation (2), similarly to FSC.

The synthetic urine was prepared by dissolving 25.0 g of urea, 9.0 g of sodium chloride, 2.5 g of sodium phosphate, 3.0 g of ammonium chloride, and 3.0 g of sodium sulfite in 750 mL of deionized water in a volumetric flask. Subsequently, the final volume was adjusted to 1.0 L [104,121]. The buffers were prepared in accordance with AAT Bioquest procedures [122].

#### 4.3.8. Synthetic Urine Retention Capacity

The retention capacity of synthetic urine by the hydrogels was evaluated over an 8 h period. The initial swollen mass of hydrogels was measured at equilibrium swelling (m_eq_) in synthetic urine. Mass measurements were subsequently obtained at predetermined time intervals (m_t_). The retention capacity was calculated using the following equation [109]:

Retention Capacity (%) = (m_t_/m_eq_) × 100 (3)

#### 4.3.9. Absorbency Under Load

The absorbency under load (AUL) procedure was designed to assess the water absorption capacity of hydrogels under a specific applied load [102]. In this procedure, a 60 mm glass filter plate was positioned in a tray, and a 60 mm diameter filter paper was placed on the plate. A pre-weighed dried hydrogel sample (W_dry_) was positioned on filter paper, and a cylindrical beaker (Ø = 60 mm) with a weight was used to apply a load of 50 g.cm^−2^ to the sample. The tests were conducted with synthetic urine, ensuring that the fluid completely covered the filter paper and reached the upper surface of the filter plate. After one hour, the sample was reweighed to determine the amount of fluid absorbed (W_abs_). The absorbency under load (AUL) was subsequently calculated using the following equation [106]:

AUL (g/g) = (W_abs_ − W_dry_)/W_dry_ (4)

#### 4.3.10. Centrifuge Retention Capacity

The centrifuge retention capacity (CRC) method was designed to access the ability of hydrogels to retain fluids after being saturated and centrifuged under controlled conditions [106]. The procedure was adapted from ISO 17190-6:2020 Urine-absorbing aids for incontinence—Polyacrylate superabsorbent powders. Part 6: Test method for determination of the fluid retention capacity in saline solution by gravimetric measurement following centrifugation. In brief, a predetermined quantity of synthetic urine swollen hydrogel (100 mg) was subjected to centrifugation at a g-force of 250 G (1400 rpm) for 3 min [123]. The excess fluid was carefully removed, and the samples were subsequently reweighed. The CRC was calculated according to [123]

CRC (g/g) = (W_c_ − W_eq_)/W_eq_ (5)

where *W_c_* is the weight of the hydrogel after centrifugation, and *W_eq_* is the initial weight of the full swollen hydrogel.

#### 4.3.11. Cytotoxicity Evaluation

The hydrogels were placed on a 6-well plate and sterilized for 25 min on each side with UV light irradiation prior to in vitro assays. To evaluate the impact of hydrogels on cell viability, Normal Human Dermal Fibroblasts (NHDF) cells were dispersed on the surface of each sample at a density of 1 × 10^5^ per well. The cell-seeded hydrogels were maintained at 37 °C and 5% CO_2_ for 1, 3, and 7 days. After the incubation time points, the medium was removed, and 10% alamarBlue™ HS Cell Viability Reagent (ThermoFisher Scientific) in MEM Alpha modification with L-glutamine, ribo- and deoxyribonucleosides (HyClone) was added to each well, followed by incubation at 37 °C in an incubator for 4 h. Then, 100 μL (in triplicate) of solution from each well was placed in a 96-well plate, and the absorbance at 570 nm was determined in a microplate reader (Bio-Tek Instrument, Inc., Winooski, VT, USA). Untreated NHDF cells, i.e., cells seeded directly over the surface of the wells without hydrogels, were used as controls. Cell viability was calculated as the percentage of survival relative to untreated NHDF cells, which were considered to have 100% viability [124].

#### 4.3.12. Statistical Analysis

All quantitative data were obtained from at least three parallel samples and are expressed as mean ± standard deviations (SD). Significant differences among each experimental group were determined through two-way analysis of variance (ANOVA), followed by Tukey’s multiple comparisons test. A *p*-value lower than 0.05 (**** *p* < 0.0001) was considered to be statistically significant. Data analysis was performed in GraphPad Prism version 10.4.0 Software (GraphPad Software Inc., La Jolla, CA, USA).

## Figures and Tables

**Table 1:** Homopolymers hydrogels prepared from the proposed monomers ^a^. Maximum swelling capacity evaluated at 24 h.

Sample ^b^	Monomer	Crosslinker ^c^ (% *w*/*w*)	Hydrogel Formation ^d^	Maximum Swelling (g/g)	Gel Content (% *w*/*w*)
P_c1	PAHEMA (P)	1.0	No	-	-
P_c3		3.0	No	-	-
P_c5		5.0	Yes	19.6 ± 1.5	31.1 ± 1.6
S_c1	SPM (S)	1.0	No	-	-
S_c3		3.0	Yes	321.5 ± 20.8	52.0 ± 2.2
S_c5		5.0	Yes	272.9 ± 19.0	76.7 ± 2.7
M_c1	METAC (M)	1.0	No	-	-
M_c3		3.0	Yes	242.6 ± 26.2	33.2 ± 2.6
M_c5		5.0	Yes	115.1 ± 7.0	74.3 ± 1.4

^a^ Homopolymers hydrogels were prepared in a solution of 10% monomer concentration, and 1% of UV initiator. ^b^ Samples were labeled as X_c*y*, where X represents the initial letter of monomer abbreviation and *y* the amount of crosslinking agent; ^c^ crosslinker used was BAAm; ^d^ when no hydrogel was formed, only a more viscous solution was obtained.

**Table 2:** Formulations of the prepared hydrogels to evaluate the effects of monomers (gray shaded) in cellulose hydrogels. All hydrogels were prepared with 1% *w*/*w* of crosslinker, and 1% *w*/*w* of UV-initiator. Maximum swelling was evaluated at 24 h. The presented values represent the mean of three samples. The most promising results were highlighted (green shaded).

Sample ^a^	AC:Monomers Ratio ^b^ (% *w*/*w*)	Monomers Ratio ^c^ (%w_monomers_/w)			Maximum Swelling (g/g)	Gel Content (% *w*/*w*)
		PAHEMA	SPM	METAC		
Cel_100_	100	-	-	-	8.4 ± 0.4	97.7 ± 0.9
Cel_50__P_50_	50:50	50	-	-	13.2 ± 0.4	77.7 ± 3.0
Cel_50__S_50_		-	50	-	15.9 ± 1.4	87.6 ± 1.5
Cel_50__M_50_		-	-	50	25.7 ± 0.9	89.1 ± 2.9
Cel_50__P_25__S_25_		25	25	-	16.2 ± 0.4	79.0 ± 1.5
Cel_50__P_25__M_25_		25	-	25	12.3 ± 0.7	79.0 ± 2.2
Cel_50__S_25__M_25_		-	25	25	14.2 ± 0.6	77.9 ± 1.3
Cel_50__P_16.7__S_16.7__M_16.7_		16.7	16.7	16.7	29.1 ± 1.3	81.7 ± 0.7
Cel_50__P_25__S_12.5__M_12.5_		25	12.5	12.5	19.0 ± 0.6	71.3 ± 1.0
Cel_50__P_12.5__S_25__M_12.5_		12.5	25	12.5	22.2 ± 0.5	87.2 ± 4.8
Cel_50__P_12.5__S_12.5__M_25_		12.5	12.5	25	22.2 ± 0.9	76.9 ± 1.5

^a^ Samples were labeled as Cel_a__P_b__S_c__M_d_, where P, S, M represents the initial letters of each monomer abbreviation, and a, b, c, and d denote the mass ratio of each component in the formulation; ^b^ AC derivative and monomers mass ratio in the formulation; ^c^ monomers mass ratio in formulation.

**Table 3:** Formulations of the prepared hydrogels to study the crosslinker amount (gray shaded) in cellulose hydrogels. All hydrogels were prepared with a AC/monomers mass ratio of 50:50, and 1.0% *w*/*w* of UV-initiator. Maximum swelling was evaluated at 24 h. The presented values represent the mean of the three samples. The most promising results are highlighted (green shaded).

Sample ^a^	Monomers Ratio ^b^ (% *w*/*w*_monomers_)			Crosslinker Amount (% *w*/*w*)	Maximum Swelling (g/g)	Gel Content (% *w*/*w*)
	PAHEMA	SPM	METAC			
Cel_50__P_16.7__S_16.7__M_16.7_	16.7	16.7	16.7	1.0 ^c^	29.1 ± 1.3	81.7 ± 0.7
				0.5	29.4 ± 1.0	80.2 ± 1.3
				0.25	18.0 ± 1.9	56.2 ± 3.3
				0.1	9.1 ± 1.0	50.9 ± 1.4
Cel_50__P_12.5__S_25__M_12.5_	12.5	25	12.5	1.0 ^c^	22.2 ± 0.5	87.2 ± 4.8
				0.5	22.4 ± 0.8	86.0 ± 2.1
				0.25	17.5 ± 0.9	76.0 ± 2.8
				0.1	16.2 ± 0.8	57.4 ± 1.2

^a^ Samples were labeled as Cel_a__P_b__S_c__M_d_, where P, S, M represents the initial letters of each monomer abbreviation, and a, b, c, and d denote the mass ratio of each component in the formulation; ^b^ monomers mass ratio in formulation; ^c^ amount of crosslinker agent used in previous study, values of swelling and gel content for reference.

**Table 4:** Formulations of the prepared hydrogels to study the initiator quantity (gray shaded) in cellulose hydrogels. All hydrogels were prepared with an AC/monomers mass ratio of 50:50, and 0.5% *w*/*w* of crosslinker. Maximum swelling was evaluated at 24 h. The presented values represent the mean of three samples. The most promising results were highlighted (green shaded).

Sample ^a^	Monomers Ratio ^b^ (% *w*/*w*_monomers_)			Initiator Quantity (% *w*/*w*)	Maximum Swelling (g/g)	Gel Content (% *w*/*w*)
	PAHEMA	SPM	METAC			
Cel_50__P_16.7__S_16.7__M_16.7_	16.7	16.7	16.7	1.00 ^c^	29.4 ± 1.0	80.2 ± 1.3
				0.75	50.0 ± 6.7	65.8 ± 2.7
				0.50	52.7 ± 10.5	67.1 ± 3.0
				0.25	60.5 ± 3.5	34.0 ± 3.6
Cel_50__P_12.5__S_25__M_12.5_	12.5	25	12.5	1.00 ^c^	22.4 ± 0.8	86.0 ± 2.1
				0.75	22.6 ± 2.3	80.7 ± 1.3
				0.50	38.2 ± 5.4	81.2 ± 2.2
				0.25	92.8 ± 8.4	42.5 ± 1.6

^a^ Samples were labeled as Cel_a__P_b__S_c__M_d_, where P, S, M represents the initial letters of each monomer abbreviation, and a, b, c, and d denote the mass ratio of each component in the formulation; ^b^ monomers mass ratio in formulation; ^c^ amount of initiator used in previous studies, values of swelling and gel content for reference.

**Table 5:** Formulations of the prepared hydrogels to evaluate the influence of the AC/monomers mass ratio (gray shaded) in cellulose hydrogels. All hydrogels were prepared with 0.5% *w*/*w* of crosslinker, and 0.5% *w*/*w* of initiator. Maximum swelling was evaluated at 24 h. The presented values represent the mean of three samples. The most promising results are highlighted (green shaded).

Sample ^a^	AC:Monomers Ratio (% *w*/*w*)	Monomers Ratio ^b^ (% *w*/*w*_monomers_)			Maximum Swelling (g/g)	Gel Content (% *w*/*w*)
		PAHEMA	SPM	METAC		
Cel_50__P_16.7__S_16.7__M_16.7_	50:50 ^c^	16.7	16.7	16.7	52.7 ± 10.5	67.1 ± 3.0
Cel_25__P_25__S_25__M_25_	25:75	25	25	25	53.2 ± 0.9	47.6 ± 1.4
Cel_10__P_30__S_30__M_30_	10:90	30	30	30	94.6 ± 2.9	38.5 ± 1.1
Cel_50__P_12.5__S_25__M_12.5_	50:50 ^c^	12.5	25	12.5	38.2 ± 5.4	81.2 ± 2.2
Cel_25__P_18.8__S_37.5__M_18.8_	25:75	18.75	37.5	18.75	37.8 ± 1.7	70.8 ± 2.2
Cel_10__P_22.5__S_45__M_22.5_	10:90	22.5	45	22.5	102.3 ± 6.5	49.2 ± 2.4

^a^ Samples were labeled as Cel_a__P_b__S_c__M_d_, where P, S, M represents the initial letters of each monomer abbreviation, and a, b, c, and d denote the mass ratio of each component in the formulation; ^b^ monomers mass ratio in formulation; ^c^ AC/monomers mass ratio used in previous studies, values of swelling and gel content for reference.

**Table 6:** Most promising cellulose-based hydrogels formulations. The higher swelling values obtained, the mass ration between AC/monomers, and gel content effect were studied regarding these formulations’ variations.

Sample ^a^	AC/Monomers Ratio ^b^ (% *w*/*w*)	Monomers Ratio ^c^ (% *w*/*w*_monomers_)			Crosslinker Amount ^e^ (% *w*/*w*)	Initiator Quantity ^f^ (% *w*/*w*)
		PAHEMA	SPM	METAC		
Cel_100_	100:0	-	-	-	0.5	0.5
Cel_50__P_12.5__S_25__M_12.5_	50:50	12.5	25	12.5		0.5
Cel_50__P_12.5__S_25__M_12.5__i0.25 ^d^	50:50	12.5	25	12.5		0.25
Cel_10__P_22.5__S_45__M_22.5_	10:90	22.5	45	22.5		0.5

^a^ Samples were labeled as Cel_a__P_b__S_c__M_d_, where P, S, M represents the initial letters of each monomer abbreviation, and a, b, c, and d denote the mass ratio of each component in the formulation; ^b^ AC and monomers mass ratio in the formulation; ^c^ monomers mass ratio in formulation; ^d^ the i0.25 was introduced in this sample label to identify the lower initiator amount used in its preparation (i, from initiator, and 0.25 the corresponding amount used), and was studied because was the higher swelling achieved in a 50:50 AC/monomers ratio; ^e^ the crosslinker used was N,N’-methylenebisacrylamide; ^f^ the initiator used was 2-hydroxy-2-methylpropiophenone.

## Data Availability

The authors confirm that supporting information is available within the article.

## Supplementary Materials

The following supporting information can be downloaded at: https://www.mdpi.com/article/10.3390/gels11040282/s1, Figure S1: Allyl cellulose DS calculation by ^1^H NMR spectra.; Figure S2: ^13^C NMR spectra of allyl cellulose recorded in D_2_O.; Figure S3: HSQC NMR spectra of allyl cellulose recorded in D_2_O. Table S1: Thermogravimetric properties of cellulose powder, AC derivative, prepared hydrogels, and SAP from baby diaper.; Figure S4: Cel_10__P_22.5__S_45__M_22.5_ hydrogel dried samples prepared by: A—cutting hydrogels into 20 mm diameter disks, B—cryogenically fracturing the hydrogels using a mortar and pestle, C—grinding the hydrogels using a coffee grinder, and D—manually broken into coarse pieces.

## References

[1] Bashari A., Rouhani Shirvan A., Shakeri M. (2018). Cellulose-Based Hydrogels for Personal Care Products. Polym. Adv. Technol..

[2] Perez M.V., Navarro P.X.S., Morillas A.V., Valdemar R.M.E., Araiza J.P.H.L. (2021). Waste Management and Environmental Impact of Absorbent Hygiene Products: A Review. Waste Manag. Res..

[3] Bae J., Kwon H., Kim J. (2018). Safety Evaluation of Absorbent Hygiene Pads: A Review on Assessment Framework and Test Methods. Sustainability.

[4] Jyoti D., Sinha R. (2023). Physiological Impact of Personal Care Product Constituents on Non-Target Aquatic Organisms. Sci. Total Environ..

[5] Ntekpe M., Mbong E., Mbong O., Edem E., Edem N., Hussain S. (2020). Disposable Diapers: Impact of Disposal Methods on Public Health and the Environment. Am. J. Med. Public Health.

[6] Płotka-Wasylka J., Makoś-Chełstowska P., Kurowska-Susdorf A., Treviño M.J.S., Guzmán S.Z., Mostafa H., Cordella M. (2022). End-of-Life Management of Single-Use Baby Diapers: Analysis of Technical, Health and Environment Aspects. Sci. Total Environ..

[7] Ismaeilimoghadam S., Jonoobi M., Hamzeh Y., Azimi B., Mezzetta A., Guazzelli L., Cinelli P., Seggiani M., Danti S. (2024). Development and Characterization of Sodium Alginate-Based Bio-Hybrid Super Absorbent Polymer with High Retention Capacity Suitable for Baby Diapers. J. Polym. Environ..

[8] Demichelis F., Martina C., Fino D., Tommasi T., Deorsola F.A. (2023). Life Cycle Assessment of Absorbent Hygiene Product Waste: Evaluation and Comparison of Different End-of-Life Scenarios. Sustain. Prod. Consum..

[9] Tsigkou K., Zagklis D., Vasileiadi A., Kostagiannakopoulou C., Sotiriadis G., Anastopoulos I., Kostopoulos V., Zafiri C., Kornaros M. (2022). Used Disposable Nappies: Environmental Burden or Resource for Biofuel Production and Material Recovery?. Resour. Conserv. Recycl..

[10] Carlucci G. (2012). New Technologies for Feminine Hygiene Products with Reduced Environmental Impact. Proceedings of the WIT Transactions on Ecology and the Environment.

[11] Yang Y., Liang Z., Zhang R., Zhou S., Yang H., Chen Y., Zhang J., Yin H., Yu D. (2024). Research Advances in Superabsorbent Polymers. Polymers.

[12] Haque M.O., Mondal M.I.H., Mondal M.I.H. (2018). Cellulose-Based Hydrogel for Personal Hygiene Applications. Cellulose-Based Superabsorbent Hydrogels.

[13] Zhang W., Wang P., Liu S., Chen J., Chen R., He X., Ma G., Lei Z. (2021). Factors Affecting the Properties of Superabsorbent Polymer Hydrogels and Methods to Improve Their Performance: A Review. J. Mater. Sci..

[14] Ma X., Wen G. (2020). Development History and Synthesis of Super-Absorbent Polymers: A Review. J. Polym. Res..

[15] Buchholz F.L. (1996). Superabsorbent Polymers: An Idea Whose Time Has Come. J. Chem. Educ..

[16] Darbre P.D. (2023). Chapter 1-Introduction to Personal Care Products. Personal Care Products and Human Health.

[17] Patiño-Masó J., Serra-Parareda F., Tarrés Q., Mutjé P., Espinach F.X., Delgado-Aguilar M. (2019). TEMPO-Oxidized Cellulose Nanofibers: A Potential Bio-Based Superabsorbent for Diaper Production. Nanomaterials.

[18] Kabir S.M.F., Sikdar P.P., Haque B., Bhuiyan M.A.R., Ali A., Islam M.N. (2018). Cellulose-Based Hydrogel Materials: Chemistry, Properties and Their Prospective Applications. Prog. Biomater..

[19] Sannino A., Demitri C., Madaghiele M. (2009). Biodegradable Cellulose-Based Hydrogels: Design and Applications. Materials.

[20] Bhaladhare S., Das D. (2022). Cellulose: A Fascinating Biopolymer for Hydrogel Synthesis. J. Mater. Chem. B.

[21] Klemm D., Heublein B., Fink H.P., Bohn A. (2005). Cellulose: Fascinating Biopolymer and Sustainable Raw Material. Angew. Chem. Int. Ed..

[22] Qureshi M.A., Nishat N., Jadoun S., Ansari M.Z. (2020). Polysaccharide Based Superabsorbent Hydrogels and Their Methods of Synthesis: A Review. Carbohydr. Polym. Technol. Appl..

[23] Zou P., Yao J., Cui Y.-N., Zhao T., Che J., Yang M., Li Z., Gao C. (2022). Advances in Cellulose-Based Hydrogels for Biomedical Engineering: A Review Summary. Gels.

[24] Kundu R., Mahada P., Chhirang B., Das B. (2022). Cellulose Hydrogels: Green and Sustainable Soft Biomaterials. Curr. Res. Green Sustain. Chem..

[25] Berradi A., Aziz F., Achaby M.E., Ouazzani N., Mandi L. (2023). A Comprehensive Review of Polysaccharide-Based Hydrogels as Promising Biomaterials. Polymers.

[26] Chang C., Zhang L. (2011). Cellulose-Based Hydrogels: Present Status and Application Prospects. Carbohydr. Polym..

[27] Liu T., Chen W., Li K., Long S., Li X., Huang Y. (2023). Toughening Weak Polyampholyte Hydrogels with Weak Chain Entanglements via a Secondary Equilibrium Approach. Polymers.

[28] Toleutay G., Su E., Yelemessova G. (2023). Equimolar Polyampholyte Hydrogel Synthesis Strategies with Adaptable Properties. Polymers.

[29] Haag S.L., Bernards M.T. (2017). Polyampholyte Hydrogels in Biomedical Applications. Gels.

[30] Arredondo R., Yuan Z., Sosa D., Johnson A., Beims R.F., Li H., Wei Q., Xu C.C. (2023). Performance of a Novel, Eco-Friendly, Cellulose-Based Superabsorbent Polymer (Cellulo-SAP): Absorbency, Stability, Reusability, and Biodegradability. Can. J. Chem. Eng..

[31] Omidian H., Akhzarmehr A., Chowdhury S.D. (2024). Advancements in Cellulose-Based Superabsorbent Hydrogels: Sustainable Solutions across Industries. Gels.

[32] Qi H., Chang C., Zhang L. (2008). Effects of Temperature and Molecular Weight on Dissolution of Cellulose in NaOH/Urea Aqueous Solution. Cellulose.

[33] Paula C.T.B., Rebelo R.C., Coelho J., Serra A.C. (2019). The Impact of the Introduction of Hydrolyzed Cellulose on the Thermal and Mechanical Properties of LDPE Composites. Eur. J. Wood Wood Prod..

[34] Setu M.N.I., Mia M.Y., Lubna N.J., Chowdhury A.A. (2015). Preparation of Microcrystalline Cellulose from Cotton and Its Evaluation as Direct Compressible Excipient in the Formulation of Naproxen Tablets. Dhaka Univ. J. Pharm. Sci..

[35] Qi H., Liebert T., Heinze T. (2012). Homogenous Synthesis of 3-Allyloxy-2-Hydroxypropyl-Cellulose in NaOH/Urea Aqueous System. Cellulose.

[36] Tong R., Chen G., Tian J., He M. (2020). Highly Transparent, Weakly Hydrophilic and Biodegradable Cellulose Film for Flexible Electroluminescent Devices. Carbohydr. Polym..

[37] Tong R., Chen G., Pan D., Qi H., Li R., Tian J., Lu F., He M. (2019). Highly Stretchable and Compressible Cellulose Ionic Hydrogels for Flexible Strain Sensors. Biomacromolecules.

[38] Silva R., Rebelo R.C., Paula C.T.B., Pereira P., Fonseca A.C., Serra A.C., Coelho J.F.J. (2025). All-Cellulose Resin for 3D Printing Hydrogels via Digital Light Processing (DLP). Int. J. Biol. Macromol..

[39] Moreira R., Rebelo R.C., Coelho J.F.J., Serra A.C. (2024). Novel Thermally Regenerated Flexible Cellulose-Based Films. Eur. J. Wood Wood Prod..

[40] Ribeiro D.C.M., Rebelo R.C., De Bon F., Coelho J.F.J., Serra A.C. (2021). Process Development for Flexible Films of Industrial Cellulose Pulp Using Superbase Ionic Liquids. Polymers.

[41] Rebelo R.C., Ribeiro D.C.M., Pereira P., De Bon F., Coelho J.F.J., Serra A.C. (2023). Cellulose-Based Films with Internal Plasticization with Epoxidized Soybean Oil. Cellulose.

[42] Tong R., Chen G., Tian J., He M. (2019). Highly Stretchable, Strain-Sensitive, and Ionic-Conductive Cellulose-Based Hydrogels for Wearable Sensors. Polymers.

[43] Kim J.S., Kim D.H., Lee Y.S. (2021). The Influence of Monomer Composition and Surface-CrossLinking Condition on Biodegradation and Gel Strength of Super Absorbent Polymer. Polymers.

[44] Mallik A.K., Shahruzzaman M., Sakib M.N., Zaman A., Rahman M.S., Islam M.M., Islam M.S., Haque P., Rahman M.M., Mondal M.I.H. (2019). Benefits of Renewable Hydrogels over Acrylate and Acrylamide-Based Hydrogels BT. Cellulose-Based Superabsorbent Hydrogels.

[45] Sennakesavan G., Mostakhdemin M., Dkhar L.K., Seyfoddin A., Fatihhi S.J. (2020). Acrylic Acid/Acrylamide Based Hydrogels and Its Properties—A Review. Polym. Degrad. Stab..

[46] Klaunig J.E. (2008). Acrylamide Carcinogenicity. J. Agric. Food Chem..

[47] Besaratinia A., Pfeifer G.P. (2007). A Review of Mechanisms of Acrylamide Carcinogenicity. Carcinogenesis.

[48] Beland F.A., Mellick P.W., Olson G.R., Mendoza M.C.B., Marques M.M., Doerge D.R. (2013). Carcinogenicity of Acrylamide in B6C3F1 Mice and F344/N Rats from a 2-Year Drinking Water Exposure. Food Chem. Toxicol..

[49] McLaughlin J.E., Parno J., Garner F.M., Clary J.J., Thomas W.C., Murphy S.R. (1995). Comparison of the Maximum Tolerated Dose (MTD) Dermal Response in Three Strains of Mice Following Repeated Exposure to Acrylic Acid. Food Chem. Toxicol..

[50] Vodička P., Gut I., Frantík E. (1990). Effects of Inhaled Acrylic Acid Derivatives in Rats. Toxicology.

[51] Lugović-Mihić L., Filija E., Varga V., Premuž L., Parać E., Tomašević R., Barac E., Špiljak B. (2024). Unwanted Skin Reactions to Acrylates: An Update. Cosmetics.

[52] Nikolić L.B., Zdravković A.S., Nikolić V.D., Ilić-Stojanović S.S., Mondal M.I.H. (2019). Synthetic Hydrogels and Their Impact on Health and Environment. Cellulose-Based Superabsorbent Hydrogels.

[53] Kemal E., Adesanya K.O., Deb S. (2011). Phosphate Based 2-Hydroxyethyl Methacrylate Hydrogels for Biomedical Applications. J. Mater. Chem..

[54] Guven M.N., Balaban B., Demirci G., Yagci Acar H., Okay O., Avci D. (2021). Bisphosphonate-Functionalized Poly(Amido Amine) Crosslinked 2-Hydroxyethyl Methacrylate Hydrogel as Tissue Engineering Scaffold. Eur. Polym. J..

[55] Paripovic D., Hall-Bozic H., Klok H.-A. (2012). Osteoconductive Surfaces Generated from Peptide Functionalized Poly(2-Hydroxyethyl Methacrylate-Co-2-(Methacryloyloxy)Ethyl Phosphate) Brushes. J. Mater. Chem..

[56] Zhang Y., Wang Y. (2013). Photopolymerization of Phosphoric Acid Ester-Based Self-Etch Dental Adhesives. Dent. Mater. J..

[57] Silano V., Barat Baviera J.M., Bolognesi C., Chesson A., Cocconcelli P.S., Crebelli R., Gott D.M., Grob K., Lambré C., EFSA Panel on Food Contact Materials, Enzymes and Processing Aids (CEP) (2020). Safety Assessment of the Substance Phosphoric Acid, Mixed Esters with 2-Hydroxyethyl Methacrylate, for Use in Food Contact Materials. EFSA J..

[58] Goncalves A.A.L., Fonseca A.C., Fabela I.G.P., Serra A.C. (2016). Synthesis and Characterization of High Performance Superabsorbent Hydrogels Using Bis[2-(Methacryloyloxy)Ethyl] Phosphate as Crosslinker. Express Polym. Lett..

[59] Romischke J., Eickner T., Grabow N., Kragl U., Oschatz S. (2024). 3-Sulfopropyl Acrylate Potassium-Based Polyelectrolyte Hydrogels: Sterilizable Synthetic Material for Biomedical Application. RSC Adv..

[60] Yu Y., Cirelli M., Li P., Ding Z., Yin Y., Yuan Y., de Beer S., Vancso G.J., Zhang S. (2019). Enhanced Stability of Poly(3-Sulfopropyl Methacrylate Potassium) Brushes Coated on Artificial Implants in Combatting Bacterial Infections. Ind. Eng. Chem. Res..

[61] Gallo M., Gámiz F. (2023). Choline: An Essential Nutrient for Human Health. Nutrients.

[62] Zeisel S.H. (2000). Choline: An Essential Nutrient for Humans. Nutrition.

[63] Li J., Song Y., Xu D., Zhang X., Du J., Liu R., Zhao R., Chen Q., Volodine A., Dewil R. (2024). Choline Chloride Modification of Nanofiltration Membranes for Improving Heavy Metal Ions Separation in Wastewater. J. Memb. Sci..

[64] Fanfoni L., Marsich E., Turco G., Breschi L., Cadenaro M. (2021). Development of Di-Methacrylate Quaternary Ammonium Monomers with Antibacterial Activity. Acta Biomater..

[65] Uka D., Kukrić T., Krstonošić V., Jović B., Kordić B., Pavlović K., Popović B.M. (2024). NADES Systems Comprising Choline Chloride and Polyphenols: Physicochemical Characterization, Antioxidant and Antimicrobial Activities. J. Mol. Liq..

[66] Song Y., Sun N., Jiang Y., Zhu H., Yu Y., Lai G., Yang X. (2024). High Hydrophilic and Antibacterial Efficient UV−Curable Silicone−Containing Choline Chloride Quaternary Ammonium Salts Functionalized Materials. Macromol. Rapid Commun..

[67] Ho T.-C., Chang C.-C., Chan H.-P., Chung T.-W., Shu C.-W., Chuang K.-P., Duh T.-H., Yang M.-H., Tyan Y.-C. (2022). Hydrogels: Properties and Applications in Biomedicine. Molecules.

[68] Bashir S., Hina M., Iqbal J., Rajpar A.H., Mujtaba M.A., Alghamdi N.A., Wageh S., Ramesh K., Ramesh S. (2020). Fundamental Concepts of Hydrogels: Synthesis, Properties, and Their Applications. Polymers.

[69] Siryk O., Goncharuk O., Samchenko Y., Kernosenko L., Szewczuk-Karpisz K. (2024). Comparison of Structural, Water-Retaining and Sorption Properties of Acrylamide-Based Hydrogels Cross-Linked by Physical and Chemical Methods. ChemPhysChem.

[70] Zhong M., Liu Y.-T., Liu X.-Y., Shi F.-K., Zhang L.-Q., Zhu M.-F., Xie X.-M. (2016). Dually Cross-Linked Single Network Poly(Acrylic Acid) Hydrogels with Superior Mechanical Properties and Water Absorbency. Soft Matter.

[71] Saraydın D., Karadagˇ E., Işıkver Y., Şahiner N., Güven O. (2004). The Influence of Preparation Methods on the Swelling and Network Properties of Acrylamide Hydrogels with Crosslinkers. J. Macromol. Sci. Part A.

[72] Pekcan Ö., Kara S. (2012). Gelation Mechanisms. Mod. Phys. Lett. B.

[73] Matsumoto A., Kumagai T., Aota H., Kawasaki H., Arakawa R. (2009). Reassessment of Free-Radical Polymerization Mechanism of Allyl Acetate Based on End-Group Determination of Resulting Oligomers by MALDI-TOF-MS Spectrometry. Polym. J..

[74] Jiang C., Zhou C., Tang W., Chen G., Yin S.-N., Xie W., Wu D. (2023). Crosslinking of Bacterial Cellulose toward Fabricating Ultrastretchable Hydrogels for Multiple Sensing with High Sensitivity. ACS Sustain. Chem. Eng..

[75] Hwang U., Moon H., Park J., Jung H.W. (2024). Crosslinking and Swelling Properties of PH-Responsive Poly(Ethylene Glycol)/Poly(Acrylic Acid) Interpenetrating Polymer Network Hydrogels. Polymers.

[76] Pruksawan S., Lim J.W.R., Lee Y.L., Lin Z., Chee H.L., Chong Y.T., Chi H., Wang F. (2023). Enhancing Hydrogel Toughness by Uniform Cross-Linking Using Modified Polyhedral Oligomeric Silsesquioxane. Commun. Mater..

[77] Nasution H., Harahap H., Dalimunthe N.F., Ginting M.H.S., Jaafar M., Tan O.O.H., Aruan H.K., Herfananda A.L. (2022). Hydrogel and Effects of Crosslinking Agent on Cellulose-Based Hydrogels: A Review. Gels.

[78] Rogovina S., Aleksanyan K., Prut E., Gorenberg A. (2013). Biodegradable Blends of Cellulose with Synthetic Polymers and Some Other Polysaccharides. Eur. Polym. J..

[79] Li Z., Chen W., Zhang L., Cheng Z., Zhu X. (2015). Fast RAFT Aqueous Polymerization in a Continuous Tubular Reactor: Consecutive Synthesis of a Double Hydrophilic Block Copolymer. Polym. Chem..

[80] Lin J.-T., Lalevee J., Cheng D.-C. (2021). A Critical Review for Synergic Kinetics and Strategies for Enhanced Photopolymerizations for 3D-Printing and Additive Manufacturing. Polymers.

[81] Tong R., Chen G., Pan D., Tian J., Qi H., Li R., Lu F., He M. (2019). Ultrastretchable and Antifreezing Double-Cross-Linked Cellulose Ionic Hydrogels with High Strain Sensitivity under a Broad Range of Temperature. ACS Sustain. Chem. Eng..

[82] Altin A., Akgun B., Sarayli Bilgici Z., Begum Turker S., Avci D. (2014). Synthesis, Photopolymerization, and Adhesive Properties of Hydrolytically Stable Phosphonic Acid-Containing (Meth)Acrylamides. J. Polym. Sci. Part A Polym. Chem..

[83] Ma X., Yun H., Wu N., Niu F., Yu J. (2021). Superior Water-Resistant Poly(2-Hydroxyethyl Methacrylate Phosphate) Flame Retardant and a Transparent, Flame-Retardant, and Biodegradable Poly(Lactide) Blend Film. ACS Appl. Polym. Mater..

[84] Mushtaq S., Ahmad N.M., Nasir H., Mahmood A., Janjua H.A. (2020). Transpicuous-Cum-Fouling Resistant Copolymers of 3-Sulfopropyl Methacrylate and Methyl Methacrylate for Optronics Applications in Aquatic Medium and Healthcare. Adv. Polym. Technol..

[85] Onder A., Ilgin P., Ozay H., Ozay O. (2020). Removal of Dye from Aqueous Medium with PH-Sensitive Poly[(2-(Acryloyloxy)Ethyl]Trimethylammonium Chloride-Co-1-Vinyl-2-Pyrrolidone] Cationic Hydrogel. J. Environ. Chem. Eng..

[86] Hussein B.M.A., Karkosh Z.S.A., Safi I.N., KarKosh A.S. (2021). Impact of Phosphate Ester Addition on the Cytotoxicity of Heat Cured Denture Base Material. Mater. Today Proc..

[87] De Bon F., Azevedo I.M., Ribeiro D.C.M., Rebelo R.C., Coelho J.F.J., Serra A.C. (2022). Scaling-Up an Aqueous Self-Degassing Electrochemically Mediated ATRP in Dispersion for the Preparation of Cellulose-Polymer Composites and Films. Polymers.

[88] Shukla N.B., Bhagat R.K., Madras G. (2013). Photo and Thermal Degradation of a Cationic Superabsorbent Polymer. Polym. Plast. Technol. Eng..

[89] Pelras T., Hofman A.H., Germain L.M.H., Maan A.M.C., Loos K., Kamperman M. (2022). Strong Anionic/Charge-Neutral Block Copolymers from Cu(0)-Mediated Reversible Deactivation Radical Polymerization. Macromolecules.

[90] Zhao B., Jiang H., Lin Z., Xu S., Xie J., Zhang A. (2019). Preparation of Acrylamide/Acrylic Acid Cellulose Hydrogels for the Adsorption of Heavy Metal Ions. Carbohydr. Polym..

[91] Liu J., Li Q., Su Y., Yue Q., Gao B., Wang R. (2013). Synthesis of Wheat Straw Cellulose-g-Poly (Potassium Acrylate)/PVA Semi-IPNs Superabsorbent Resin. Carbohydr. Polym..

[92] Hajiali F., Tajbakhsh S., Marić M. (2020). Thermal Characteristics and Flame Retardance Behavior of Phosphoric Acid-Containing Poly(Methacrylates) Synthesized by RAFT Polymerization. Mater. Today Commun..

[93] Hofman A.H., Pedone M., Kamperman M. (2022). Protected Poly(3-Sulfopropyl Methacrylate) Copolymers: Synthesis, Stability, and Orthogonal Deprotection. ACS Polym. Au.

[94] Alam M.N., Islam M.S., Christopher L.P. (2019). Sustainable Production of Cellulose-Based Hydrogels with Superb Absorbing Potential in Physiological Saline. ACS Omega.

[95] Wang Y., He G., Li Z., Hua J., Wu M., Gong J., Zhang J., Ban L., Huang L. (2018). Novel Biological Hydrogel: Swelling Behaviors Study in Salt Solutions with Different Ionic Valence Number. Polymers.

[96] Simerville J.A., Maxted W.C., Pahira J.J. (2005). Urinalysis: A Comprehensive Review. Am. Fam. Physician.

[97] Bayat M.R., Baghani M. (2021). A Review on Swelling Theories of PH-Sensitive Hydrogels. J. Intell. Mater. Syst. Struct..

[98] Furukawa N., Fujihara H. (1991). Acidity, Hydrogen Bonding and Metal Complexation of Sulfonic Acids and Derivatives. Sulphonic Acids, Esters and Their Derivatives (1991).

[99] Bastos H., Gallastegui A., de Lacalle J.L., Schaeffer N., Pringle J.M., Mecerreyes D., Pozo-Gonzalo C. (2024). Ionic Polymer Absorbents Inspired by Deep Eutectic Solvents to Recover Cobalt and Nickel. New J. Chem..

[100] Chen Y., Tan H. (2006). Crosslinked Carboxymethylchitosan-g-Poly(Acrylic Acid) Copolymer as a Novel Superabsorbent Polymer. Carbohydr. Res..

[101] Pourjavadi A., Kurdtabar M. (2007). Collagen-Based Highly Porous Hydrogel without Any Porogen: Synthesis and Characteristics. Eur. Polym. J..

[102] Reshma G., Reshmi C.R., Shantikumar V.N., Menon D. (2020). Superabsorbent Sodium Carboxymethyl Cellulose Membranes Based on a New Cross-Linker Combination for Female Sanitary Napkin Applications. Carbohydr. Polym..

[103] Bachra Y., Grouli A., Damiri F., Zhu X.X., Talbi M., Berrada M. (2022). Synthesis, Characterization, and Swelling Properties of a New Highly Absorbent Hydrogel Based on Carboxymethyl Guar Gum Reinforced with Bentonite and Silica Particles for Disposable Hygiene Products. ACS Omega.

[104] Yadav S., Illa M.P., Rastogi T., Sharma C.S. (2016). High Absorbency Cellulose Acetate Electrospun Nanofibers for Feminine Hygiene Application. Appl. Mater. Today.

[105] Lacoste C., Lopez-Cuesta J.-M., Bergeret A. (2019). Development of a Biobased Superabsorbent Polymer from Recycled Cellulose for Diapers Applications. Eur. Polym. J..

[106] Bachra Y., Grouli A., Damiri F., Bennamara A., Berrada M. (2020). A New Approach for Assessing the Absorption of Disposable Baby Diapers and Superabsorbent Polymers: A Comparative Study. Results Mater..

[107] Aizawa M., Suzuki S. (1971). Properties of Water in Macromoleular Gels. III. Dilatometric Studies of the Properties of Water in Macromolecular Gels. Bull. Chem. Soc. Jpn..

[108] Ramazani-Harandi M.J., Zohuriaan-Mehr M.J., Yousefi A.A., Ershad-Langroudi A., Kabiri K. (2006). Rheological Determination of the Swollen Gel Strength of Superabsorbent Polymer Hydrogels. Polym. Test..

[109] Rebelo R.C., Báguena B.V., Pereira P., Moreira R., Coelho J.F.J., Serra A.C. (2024). Biocompatible Cellulose-Based Superabsorbents for Personal Care Products. J. Polym. Environ..

[110] Saraiva S., Rénio F., Pereira P., Santos P., Paula C.T.B., Ramalho A., Serra A.C., Fonseca A.C. (2025). Tackling the Problem of Tendon Adhesions: Physical Barriers Prepared from α-Amino Acid-Based Poly(Ester Amide)S. Polymers.

[111] Saraiva S., Pereira P., Paula C.T., Rebelo R.C., Coelho J.F.J., Serra A.C., Fonseca A.C. (2021). Development of Electrospun Mats Based on Hydrophobic Hydroxypropyl Cellulose Derivatives. Mater. Sci. Eng. C.

[112] Paula C.T.B., Leandro A., Pereira P., Coelho J.F.J., Fonseca A.C., Serra A.C. (2024). Fast-Gelling Polyethylene Glycol/Polyethyleneimine Hydrogels Degradable by Visible-Light. Macromol. Biosci..

[113] Almeida R.O., Ramos A., Alves L., Potsi E., Ferreira P.J.T., Carvalho M.G.V.S., Rasteiro M.G., Gamelas J.A.F. (2021). Production of Nanocellulose Gels and Films from Invasive Tree Species. Int. J. Biol. Macromol..

[114] Almeida R.O., Ramos A., Kimiaei E., Österberg M., Maloney T.C., Gamelas J.A.F. (2024). Improvement of the Properties of Nanocellulose Suspensions and Films by the Presence of Residual Lignin. Cellulose.

[115] Cai J., Zhang L. (2005). Rapid Dissolution of Cellulose in LiOH/Urea and NaOH/Urea Aqueous Solutions. Macromol. Biosci..

[116] Zhou J., Zhang L. (2000). Solubility of Cellulose in NaOH/Urea Aqueous Solution. Polym. J..

[117] Naserifar S., Kuijpers P.F., Wojno S., Kádár R., Bernin D., Hasani M. (2022). In Situ Monitoring of Cellulose Etherification in Solution: Probing the Impact of Solvent Composition on the Synthesis of 3-Allyloxy-2-Hydroxypropyl-Cellulose in Aqueous Hydroxide Systems. Polym. Chem..

[118] Kleemann C., Zink J., Selmer I., Smirnova I., Kulozik U. (2020). Effect of Ethanol on the Textural Properties of Whey Protein and Egg White Protein Hydrogels during Water-Ethanol Solvent Exchange. Molecules.

[119] Paula C.T.B., Pereira P., Coelho J.F.J., Fonseca A.C., Serra A.C. (2021). Development of Light-Degradable Poly(Urethane-Urea) Hydrogel Films. Mater. Sci. Eng. C.

[120] Saraiva S., Pereira P., Santos P., Ramalho A., Serra A.C., Fonseca A.C. (2024). Electrospun Mats from α-Amino Acid Based Poly(Ester Amide)s: A Promising Material for the Prevention of Tendon Adhesions. React. Funct. Polym..

[121] Frazier L.M. (2006). Superabsorbent Nanofiber Matrices. Ph.D. Thesis.

[122] Inc., AAT Bioquest Quest Calculate^TM^—Preparation and Recipe. https://www.aatbio.com/resources/buffer-preparations-and-recipes.

[123] (2020). 2020 Urine-Absorbing Aids for Incontinence—Polyacrylate Superabsorbent Powders Part 6: Test Method for Determination of the Fluid Retention Capacity in Saline Solution by Gravimetric Measurement Following Centrifugation.

[124] Paula C.T.B., Madeira A.B., Pereira P., Branco R., Morais P.V., Coelho J.F.J., Fonseca A.C., Serra A.C. (2022). ROS-Degradable PEG-Based Wound Dressing Films with Drug Release and Antibacterial Properties. Eur. Polym. J..

