# Supplementary material for: Development of Polyampholyte Cellulose-Based Hydrogels for Diapers with Improved Biocompatibility
Source: Gels. 2025 Apr 10;11(4):282. doi: 10.3390/gels11040282 (PMC12027145; doi:10.3390/gels11040282)
Supplement: Supplementary file 1 [file gels-11-00282-s001.zip › gels-3529217-supplementary.pdf]

Supplementary Materials

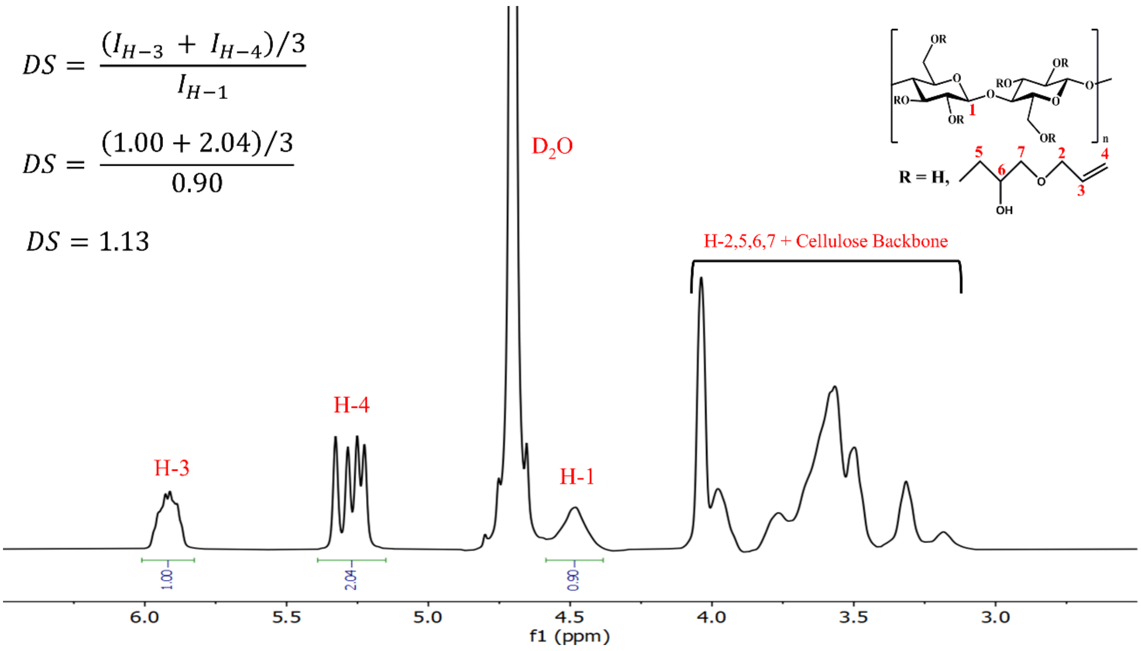

Figure S1. Allyl cellulose DS calculation by ^{1}H NMR spectra.

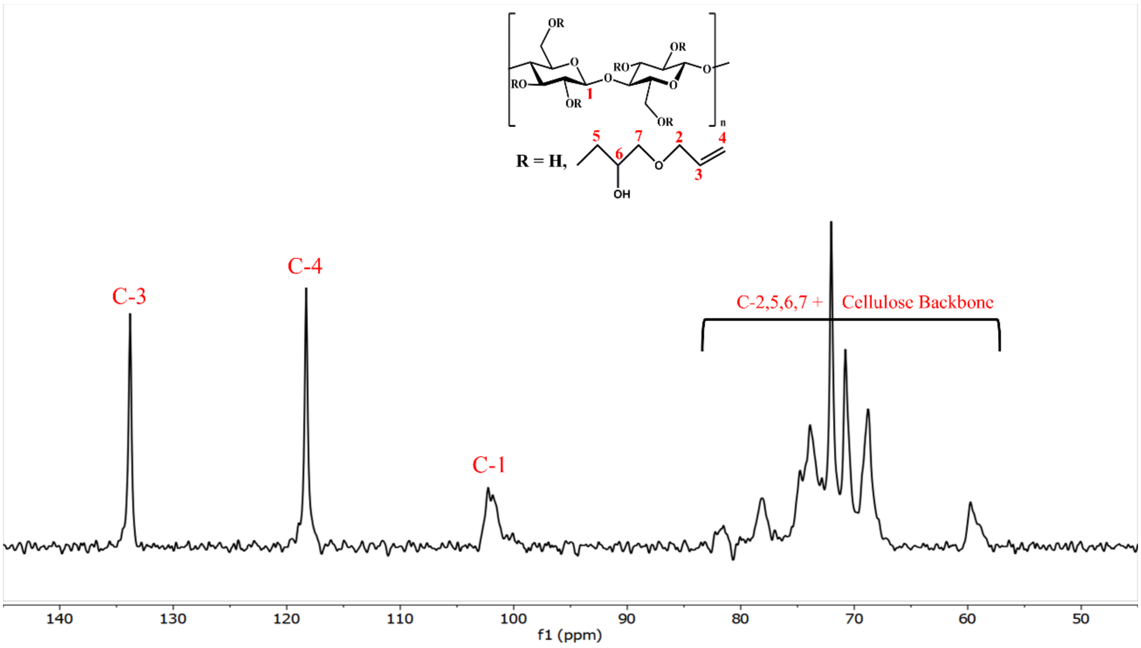

Figure S2. ^{13}C NMR spectra of allyl cellulose recorded in D_2O.

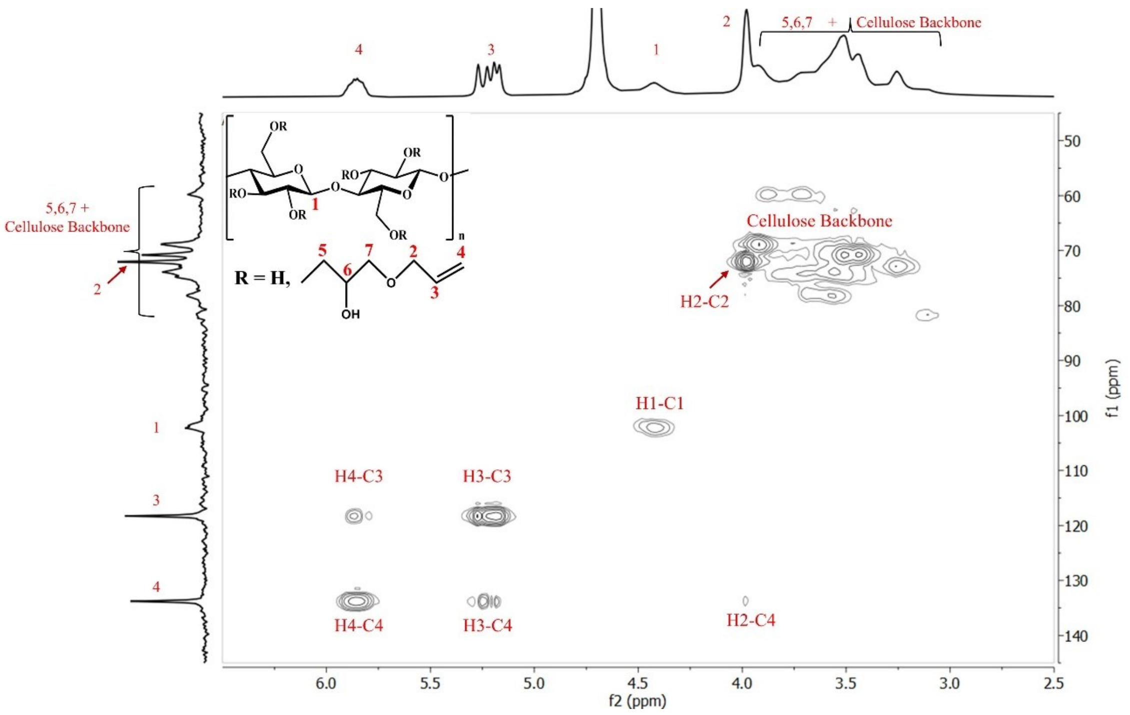

Figure S3. HSQC NMR spectra of allyl cellulose recorded in D_2O.

Table S1. Thermogravimetric properties of cellulose powder, AC derivative, prepared hydrogels, and SAP from baby diaper.

	TGA			dTG		
**Sample**	T_{95}[a] (°C)	T_{90}[b] (°C)	W_{600}[c] (°C)	$Peak_1$ (°C)	$Peak_2$ (°C)	$Peak_3$ (°C)
Cellulose powder	225.4	259.2	16.7	-	-	341.8
AC	205.1	245.1	11.1	-	239.8	340.2
Cel_{100}	234.6	275.7	13.0	-	-	330.6
Cel_{50}_$P_{12.5}$_S_{25}_$M_{12.5}$	146.0	247.5	30.1	262.1	334.3	410.2
DiaperA	91.0	147.5	41.5	109.1	362.9	456.3

[a]Temperature at 95% mass remaining; [b]Temperature at 90% mass remaining; [c]Residual mass at 600 °C.

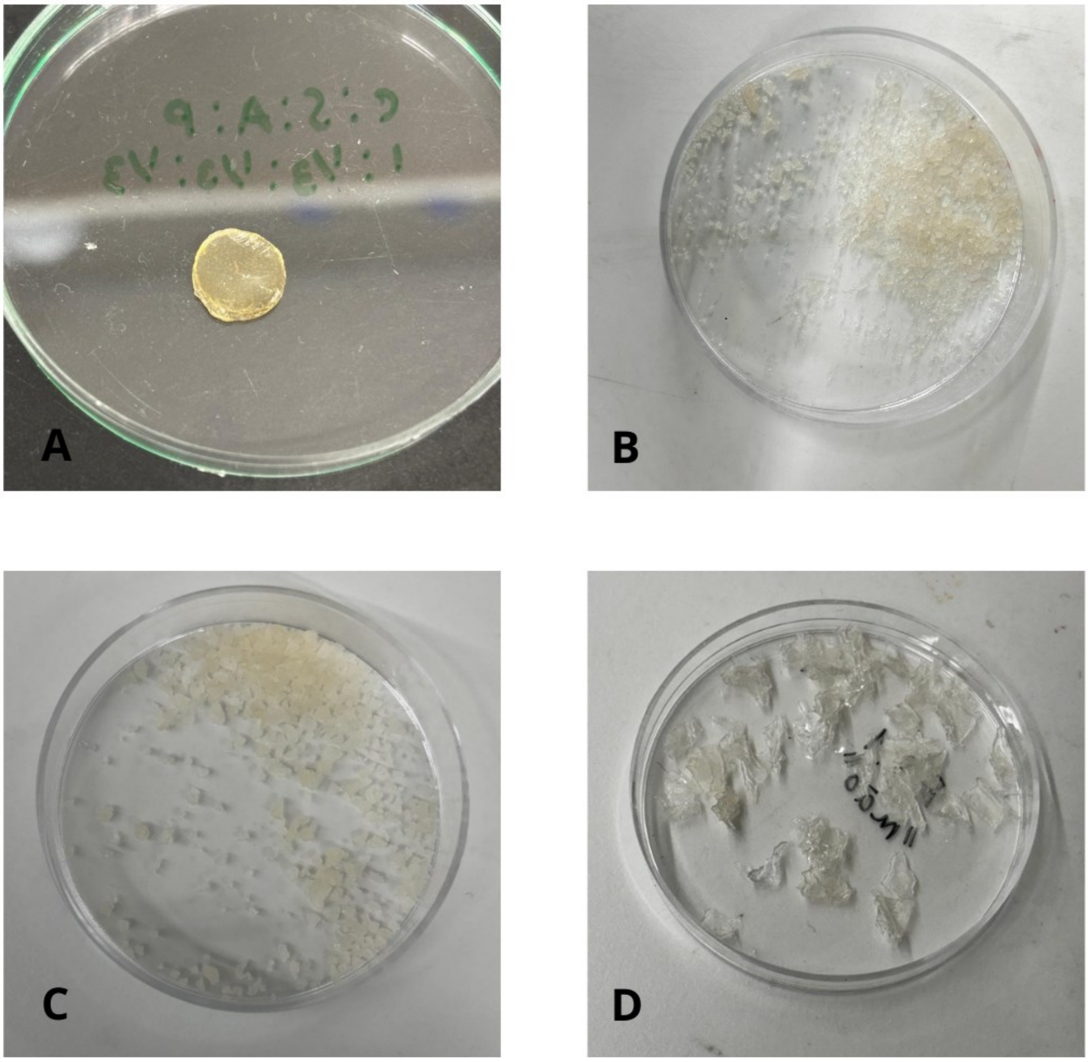

Figure S4. $Cel_{10}\_P_{22.5}\_S_{45}\_M_{22.5}$ hydrogel dried samples prepared by: A - cutting hydrogels into 20 mm diameter discs, B - cryogenically fracturing the hydrogels using a mortar and pestle, C - grinding the hydrogels using a coffee grinder, and D - manually broken into coarse pieces.
